# Advances in lanthanide probes for selective anion recognition and sensing in water

**DOI:** 10.1039/d6sc00878j

**Published:** 2026-05-22

**Authors:** Christy Siu, Stephen J. Butler

**Affiliations:** a Department of Chemistry, Loughborough University Epinal Way Loughborough LE11 3TU UK S.J.Butler@lboro.ac.uk

## Abstract

Selective anion recognition in aqueous media is an important focus in supramolecular chemistry, driven by both the intrinsic challenges of binding anions in highly competitive environments and the significant potential for applications in sensing, imaging, and sequestration. The structural diversity of anions necessitates the synthesis of host molecules with structures tailored for selective binding, overcoming high hydration energies and eliciting sensitive optical signals. Emissive lanthanide complexes, particularly those of europium(iii) and terbium(iii), continue to offer distinct advantages for anion sensing in aqueous and biological environments. Their long luminescence lifetimes enable time gating to remove background autofluorescence, while their narrow emission bands allow for precise ratiometric analysis. Variations in ligand structure and geometry can fine-tune binding selectivity and photophysical behaviour, enabling rapid and reversible anion sensing. Over the last five years, new and augmented ligand designs have enabled enhanced selectivity by controlling steric demand at the metal centre, matching host–guest charge and shape and utilising secondary non-covalent interactions for recognition. This review highlights advances over the past five years in lanthanide host design, showing how these developments have improved mechanistic understanding of binding and enabled new anion sensors and imaging probes that function in aqueous and biological media.

## Abstract 2

This review presents recent advances in lanthanide-based anion receptors, focusing on molecules with improved anion selectivity, new mechanistic insight, and real-world sensing and imaging applications.

## Graphical abstract

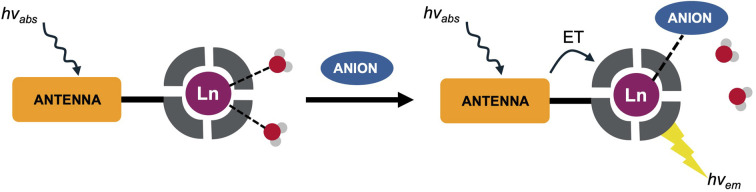

## 1. Introduction

Selective anion recognition in water remains a major challenge in supramolecular chemistry, owing to the high hydration energies of anions, their structural diversity, and the sensitivity of binding processes to pH variations in complex aqueous media.^1–5^ The past decade has seen substantial progress in anion receptor chemistry, driven by advances in synthetic host design and analytical characterisation. This has motivated the development of systems with real-world utility, including sensors and probes for biological or environmental applications. Recent reviews have highlighted how these developments have transformed anion receptor chemistry from a fundamental pursuit into a rapidly evolving field with applications in sensing, catalysis, and separation.^6–11^ The potential of synthetic receptors to monitor or modulate biological processes involving anions underscores the relevance of anion receptor chemistry. Yet, achieving selective and responsive anion recognition in aqueous conditions remains a continuing challenge that is central to advances in biological sensing, clinical diagnostics, and environmental monitoring.

Emissive lanthanide(iii) complexes, particularly those of europium(iii) and terbium(iii), represent an attractive class of anion receptors that are especially well suited for sensing applications in aqueous media.^12–14^ Their long luminescence lifetimes enable time-gated measurements that suppress short-lived background fluorescence, while their sharp line-like emission spectra permit ratiometric analysis, and their large pseudo-Stokes shifts minimise self-absorption and spectral overlap.^15–20^ Consequently, lanthanide complexes have found broad application as optical probes for sensing and imaging in biologically relevant media and in living cells, where high sensitivity and low background are essential.^21–26^ A unique feature of lanthanide ions is that their valence 4f electrons are shielded by filled 5s and 5p orbitals; as a result, they interact with anions primarily through electrostatic interactions, providing useful levels of anion affinity in water.^27^ Modifications of the surrounding ligand architecture further enable selective and reversible anion binding. These combined properties make lanthanide(iii) complexes particularly valuable for selective anion recognition in aqueous and biological environments.

Over the last 25 years, a wide range of lanthanide(iii) complexes have been developed for binding and sensing anions, as summarised in several comprehensive reviews.^12,13,21,28,29^ This article focusses on advances from the last five years, during which several highly selective lanthanide(iii) complexes have emerged. We focus on discrete, water-soluble lanthanide(iii) complexes that achieve high selectivity suitable for biological sensing, and environmental monitoring. We discuss mechanisms of anion binding and strategies to tune selectivity through variations in ligand structure and geometry, steric demand at the Ln(iii) centre, and overall complex charge. Particular emphasis is placed on europium(iii) and terbium(iii) receptors capable of: (1) detecting anions in biological media; (2) monitoring enzyme-catalysed reactions in real-time; (3) monitoring anion transport across lipid membranes; and (4) imaging anions in living cells. We also discuss probes that signal anion binding *via* circularly polarised luminescence (CPL), offering a complementary optical readout with potential for chiral or enantioselective recognition.^30–32^ Overall, this review provides an up-to-date overview of lanthanide host design principles for selective anion recognition, highlighting both the opportunities and remaining challenges in the field. Accordingly, we next outline the key design principles governing anion-selective lanthanide probes, before turning to recent representative examples.

## 2. Design considerations for lanthanide-based anion receptors

### 2.1. Preorganisation and host geometry

A preorganised lanthanide(iii) coordination environment is critical for achieving high binding affinity and selectivity in host–anion interactions. Lanthanide(iii) ions typically adopt coordination numbers between 8 and 10 in aqueous solution, and their interaction with the surrounding ligand is predominantly electrostatic in nature. Ln(iii) complexes designed for anion recognition should be kinetically inert and structurally well-defined to prevent dissociation of the Ln(iii) ion upon anion binding. This is typically achieved by using macrocyclic ligands, such as cyclen (1,4,7,10-tetraazacyclododecane) or TACN (1,4,7-triazacyclononane), or tris-bidentate tripodal scaffolds.^12,13,33^ Preorganisation of the coordination environment ensures that the binding site is suitably arranged for the target guest, leading to high-affinity and selective complex formation. This approach reflects the well-established principle that selective guest binding is optimised when the host and guest geometries are complementary.^34–36^

### 2.2. Direct Ln(iii)-anion binding and charge effects

Anion binding directly at the Ln(iii) centre typically produces the strongest interaction and can lead to pronounced photophysical responses. For such complexes, the organic ligand should provide at least one vacant coordination site, occupied by water, which can be displaced by the target anion. Electrostatic interactions play a major role in anion recognition: cationic Ln(iii) complexes preferentially bind hard oxyanions such as phosphate, bicarbonate, citrate, and fluoride, consistent with hard–soft acid–base (HSAB) theory.^14,37,38^ The overall complex charge can be tuned through the nature of the pendant donor groups, which may be anionic (*e.g.* carboxylate) or neutral (*e.g.* carbonylamide), and through peripheral ligand substituents, which may carry positive, negative, or neutral charges. Together, these design elements enable control of both the residual electropositive charge at the Ln(iii) centre and enables matching of the overall complex charge to that of the target anion.

**Fig. 1:**
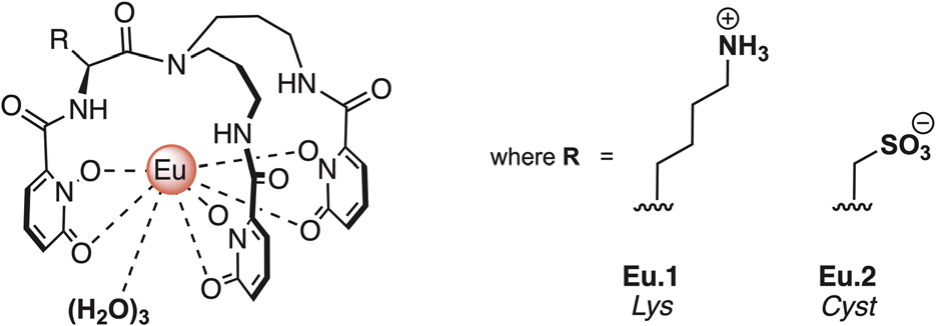
Tripodal complexes developed by Pierre and co-workers, wherein the peripheral R groups significantly influence phosphate affinity in water.

Pierre and co-workers demonstrated this principle by preparing a series of tris-bidentate Eu(iii) complexes designed to selectively bind phosphate (HPO_4_^2−^), two of which are shown in Fig. 1.^39^ The backbone R substituent was varied to introduce groups capable of hydrogen bonding, modulating the overall complex charge and increasing water solubility. The cationic complex Eu.1 displayed the highest affinity for HPO_4_^2−^ (*K*_a1_ = 4.6 × 10^5^ M^−1^, *K*_a2_ = 4.7 × 10^5^ M^−1^), attributed to direct coordination of the anion to the Eu(iii) ion supported by hydrogen bonding interactions between HPO_4_^2−^ and peripheral lysine N–H groups. Phosphate binding was accompanied by an increase in lifetime: in H_2_O, *τ* increased from 0.13 (unbound) to 0.30 ms (bound), and in D_2_O from 0.21 to 0.47 ms. In contrast, the cystic acid-derived complex Eu.2 showed no measurable affinity for HPO_4_^2−^ or other anions, highlighting the impact of introducing negatively charged groups near to the open coordination site.

### 2.3. Acid–base equilibria and pH-dependent anion affinity

An important factor in the design of Ln(iii)-based anion receptors is the acid–base chemistry of the metal centre and ligand(s) in aqueous solution. Ln(iii) ions are hard Lewis acids that polarise coordinated water molecules, resulting in relatively low p*K*_a_ values for Ln–OH_2_ deprotonation. Consequently, depending on the ligand environment, deprotonation of the coordinated water can occur near neutral pH, generating Ln–OH species that can compete directly with anion binding. For a model Ln(iii) complex containing a single inner-sphere water molecule, the first hydrolysis equilibrium can be represented as:

1 [Ln**L**(H_2_O)]^*n*+^ ⇌ [Ln**L**(OH)]^(*n*−1)+^ + H^+^

This equilibrium is important for understanding anion recognition in water, as the formation of an Eu–OH species reduces the availability of the coordination site for anion binding and alters both the charge and photophysical properties of the complex. In systems containing multiple coordinated water molecules, successive deprotonation steps further complicate speciation. At high pH, formation of insoluble Eu(OH)_3_ can occur, although this process is typically suppressed or shifted to very high pH values in well-defined macrocyclic complexes.

Ligand design thus plays a key role in modulating these equilibria. Hepta- and octadentate ligands stabilise the Ln(iii) centre and increase the effective p*K*_a_ of coordinated water. The nature of the ligand donor atoms is important here: anionic donors such as carboxylates, phosphonates, and hydroxypyridinonates (HOPO) increase overall complex stability and reduce the local electropositive character at the metal centre, whereas neutral donors such as amides or pyridines render the Ln(iii) centre more strongly electropositive and may afford lower thermodynamic stability. The balance between these donor types modulates the effective complex p*K*_a_ and the accessibility of coordination sites for anion binding.

Importantly, both the Ln(iii) receptor and the target anion are subject to pH-dependent speciation. For example, phosphate exists in approximate 1 : 1 mixture of H_2_PO_4_^−^ and HPO_4_^2−^ at neutral pH, with differing charge and thus binding affinities toward Ln(iii) ions. As such, the observed binding constants and emission changes are inherently dependent on the solution pH.^40^ Most reported Ln(iii)-based anion receptors are studied in buffered aqueous media to maintain a constant pH; however, an evaluation of anion sensing behaviour should include pH titrations of the Ln(iii) complex, ideally in the absence and presence of the anion, allowing determination of the operational pH window and the pH dependence of the emission response. For instance, Pierre demonstrated that the phosphate-selective Eu(iii) complex Eu.1 (Fig. 1) will also bind to deprotonated cyanide (CN^−^) at basic pH, which is not observed at neutral pH.^41^

These considerations ultimately define the dynamic working pH range of the Ln(iii) probe and are essential for its reliable application in complex biological or environmental media. Careful choice of buffer, including p*K*_a_ and potential metal-binding properties,^42^ is also important to avoid altering apparent binding constants or selectivity.

### 2.4. Steric modulation and anion chelation

**Fig. 2:**
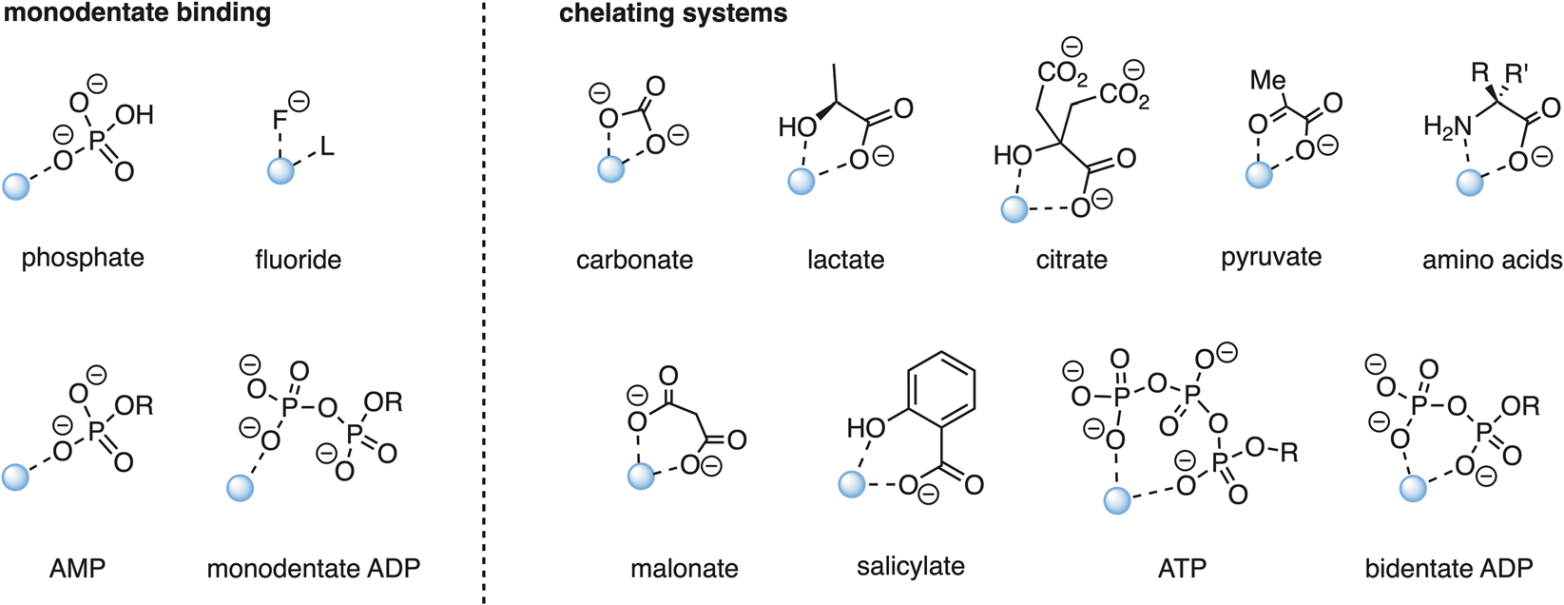
Schematic representation of anion binding modes to lanthanide(iii) complexes. Monodentate binding and bidentate chelation forming rings of varying sizes are illustrated, with examples of relevant oxyanions.

As summarised in Fig. 2, Ln(iii) ions have been shown to bind a range of monodentate and multidentate oxyanions, with the latter forming chelate rings of varying sizes and thermodynamic stabilities. Structural and spectroscopic studies, including X-ray crystallography, NMR, and emission analyses, have revealed these binding modes and provided insight into how steric constraints dictate binding preferences,^13^ and several recent examples are discussed in this review. Steric modulation at or near the anion binding pocket plays a critical role in enhancing anion discrimination by excluding non-complementary guests. Increased steric bulk near the coordination site can restrict access of larger chelating anions to the metal centre, favouring monodentate binding, whereas less encumbered environments may promote bidentate or multidentate anion coordination.^43,44^ Careful control of ligand sterics and the resulting coordination geometry thus enables tuning of anion binding. Additionally, understanding whether a lanthanide complex adopts a square antiprismatic (SAP) and/or a twisted square antiprismatic (TSAP) geometry in aqueous solution is important, as anion binding can modulate this equilibrium, leading to differences in emission spectral shape.^45^ The TSAP geometry is generally more sterically hindered, as supported by observations in Gd(iii) complexes used as contrast agents, where the TSAP isomer exhibits longer, weaker water coordination due to steric crowding around the metal centre.^46–48^

Importantly, ligands offering secondary binding motifs that combine metal coordination with hydrogen bonding, π–π stacking, or electrostatic interactions can further enhance affinity and selectivity through complementary interactions. However, incorporation of such additional binding sites can increase lipophilicity, reduce water solubility, and pose synthetic challenges. Computational methods, such as DFT, are thus increasingly employed to guide ligand design and help balance these factors effectively.^44,49–51^

### 2.5. Balancing antenna coordination and ligand rigidity

**Fig. 3:**
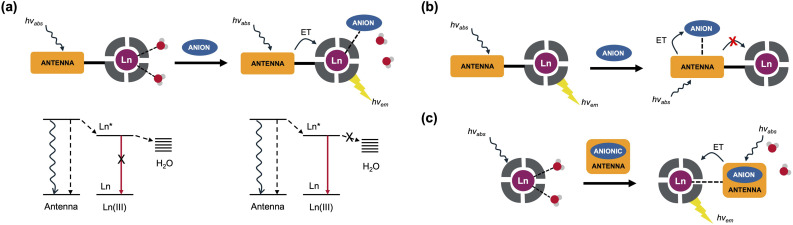
Schematic representation of different sensing mechanisms for lanthanide(iii)-based anion receptors. (a) Direct coordination of the anion to the metal centre, displacing inner-sphere water molecules, with a simplified energy diagram showing ligand-to-lanthanide energy transfer and vibrational quenching to water O–H oscillators; (b) interaction of the anion with the antenna, modulating the sensitisation process, typically resulting in luminescence quenching; (c) binding of an anion that carries an appropriate sensitiser, which “switches on” lanthanide luminescence.

To overcome the intrinsically low molar absorptivity of Ln(iii) ions, a strongly absorbing chromophore, commonly referred to as an antenna, is incorporated into the ligand structure (Fig. 3a). Upon excitation by UV (or visible) light, the antenna absorbs photon energy and efficiently transfers it to the nearby Ln(iii) centre, usually *via* their triplet excited state.^52,53^ Consequently, the positioning and coordination of the antenna chromophore become critical, as they directly influence both energy transfer efficiency and anion binding. A key requirement for efficient sensitisation is a favourable energetic gap between the antenna excited state and the accepting Ln(iii) level. In most cases, this involves a downhill energy cascade, where the donor excited state (commonly the ligand triplet state) lies higher in energy than the Ln(iii) acceptor state, typically by at least 2000 cm^−1^ at ambient temperature, to minimise thermally activated back energy transfer. For Eu(iii) and Tb(iii), this constrains the choice of antenna to those with triplet energies above approximately 19 000 and 22 000 cm^−1^, relative to their emitting levels at 17 200 and 20 400 cm^−1^ respectively.^54^ While triplet-mediated energy transfer *via* a Dexter mechanism is most common, alternative pathways can arise depending on ligand structure. For example, Eu(iii) sensitisation may proceed *via* a relaxed intramolecular charge transfer (ICT) state in systems bearing strongly electron-donating substituents,^55–57^ whereas direct singlet-state energy transfer can occur when intersystem crossing is inefficient (as observed for some coumarin chromophores).^58^ Importantly, if the antenna excited state lies too low in energy, back energy transfer can compete with Ln(iii) emission, reducing emission intensity and lifetime and introducing sensitivity to dissolved oxygen and temperature.

**Fig. 4:**
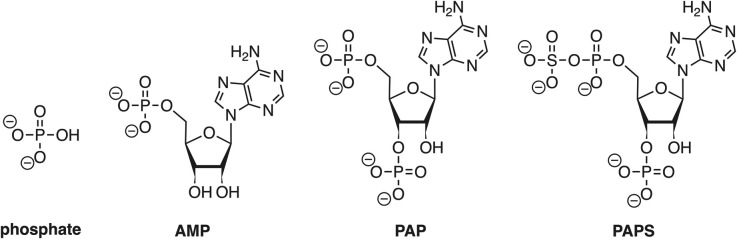
Structures of selected biologically important monophosphate anions recognised by lanthanide(iii) probes.

One common Ln(iii) probe design strategy involves direct coordination of the antenna to the lanthanide centre, often *via* a polarisable nitrogen atom of a heterocycle. This shortens the Ln–antenna distance, enables efficient sensitisation of the lanthanide excited state, and increases the rigidity of the ligand framework, helping to preorganise the anion binding site. In some cases, chelating anions compete for ligand coordination sites and displace antenna arms, altering energy transfer efficiency, emission intensity, and spectral profile.^59,60^ This displacement can be exploited as a sensitive means of signalling anion binding. When antenna displacement is undesirable, chelating strategies can be employed in which the antenna coordinates through two or more donor atoms to the Ln(iii) centre, thereby reinforcing binding and rigidity.^43,61–63^

Having established how ligand structure, sterics, and antenna positioning influence anion binding, we next consider how these interactions are translated into a measurable optical signal.

## 3. Distinct anion binding and sensing mechanisms

For anion recognition to be observed, the binding event must be translated into a measurable optical signal. Emissive europium(iii) and terbium(iii) complexes are ideally suited for this purpose. Efficient sensitisation of the lanthanide excited state is essential to achieve bright emission, while non-radiative pathways can be modulated to tune the probe response: processes that compete with antenna-to-metal energy transfer, such as photoinduced electron transfer, will affect the sensitisation efficiency, whereas direct quenching of the lanthanide excited state by inner sphere O–H/N–H oscillators will modulate the intrinsic luminescence quantum yield. By designing the ligand framework and positioning the sensitising chromophore carefully, anion binding can either enhance emission by switching off specific quenching pathways, or perturb energy transfer, each producing measurable changes in emission intensity, lifetime, or spectral shape. Anion binding and signalling at Ln(iii) centres is typically achieved through three distinct mechanisms, illustrated in Fig. 3.

### 3.1. Direct anion coordination to the Ln(iii) centre

The most common mechanism involves reversible binding of the anion to the Ln(iii) ion, displacing inner-sphere water molecules or weakly bound ligand donors (Fig. 3a). This reduces vibrational quenching pathways, leading to increased emission intensity and lifetime, and alters the coordination environment, producing changes in spectral shape. Notably, changes in the hypersensitive Δ*J* = 2 (605–630 nm) emission band of Eu(iii) compared with the relatively insensitive Δ*J* = 1 (580–605 nm) band allow ratiometric analysis, improving precision and reliability. Parker and co-workers have demonstrated this strategy effectively using a variety of heptadentate DO3A-based Eu(iii) and Tb(iii) complexes with one or two anion-binding sites. Displacement of coordinated water by anions such as lactate,^38^ HPO_4_^2−^,^14^ HCO_3_^−^,^64,65^ or citrate^66^ enhances emission intensity and lifetime, and several of these complexes have been developed into practical ratiometric assays for selective anion detection in biological fluids. Notably, for complexes in which the excited antenna can transiently reduce Eu(iii) to Eu(ii), particularly those based on overall neutral ligands lacking charged pendant donors, ligand-to-metal electron transfer can compete significantly with emission. In such systems, coordination of hard anions and displacement of water can further enhance emission by stabilising Eu(iii) against reduction and suppressing photoinduced electron transfer (PeT).^67–69^ PeT refers to an excited-state quenching pathway in which electron transfer from the excited antenna to Ln(iii) competes with radiative decay, leading to a reduction in the overall luminescence quantum yield.

### 3.2. Interaction with the antenna

Anions can interact directly with the ligand antenna *via* hydrogen bonding, electrostatic interactions, or π–π stacking, modulating the antenna singlet or triplet state (Fig. 3b). Energy or electron transfer from the anion to the antenna typically quenches luminescence by competing with ligand-to-metal energy transfer, either through dynamic exciplex formation or collisional processes. These interactions are generally weaker than direct Ln(iii) coordination and are therefore better suited for detecting anions at higher concentrations. Key examples include Eu(iii) and Tb(iii) complexes with tetraazatriphenylene or phenanthridine sensitisers, where certain anion–antenna interactions modulate the antenna excited state, resulting in luminescence quenching.^70–73^

### 3.3. Anion-sensitised emission ‘switch-on’

Less commonly, an anion bearing an appropriate sensitising group can activate lanthanide emission by transferring energy to the Ln(iii) centre upon binding (Fig. 3c). This mechanism requires the anion's triplet excite state to match the Ln(iii) accepting level and can involve direct coordination to the metal or close association *via* electrostatic interactions, positioning the sensitiser for efficient energy transfer. This strategy has been demonstrated, for example, in Tb(iii)–bisZn(ii) complexes for selective recognition of guanosine monophosphate (GMP).^74^

These three strategies form the conceptual basis for most Ln(iii)-based anion receptors. Direct anion coordination to the Ln(iii) centre is the most utilised approach, because it enables ratiometric sensing and produces distinct ‘fingerprint’ emission responses due to coordination geometry changes unique to the binding mode of each anion. However, as noted previously, anion affinity and selectivity are frequently pH-dependent, requiring careful evaluation under buffered aqueous conditions and, ideally, across multiple pH values. Interactions of the anion with the antenna provide a complementary strategy, wherein weaker non-covalent interactions, such as π–π stacking or hydrogen bonding, can modulate the antenna excited state. This typically results in overall emission changes rather than alterations to the spectral profile, making this approach more suitable for ‘switch-off’ sensors and the detection of higher anion concentrations, though generally offering lower affinity. Finally, anion-sensitised ‘switch-on’ systems can provide high selectivity for anions capable of efficiently transferring excited-state energy to the lanthanide, such as aromatic anions (*e.g.* GMP), but are more limited in scope, as many inorganic and organic anions do not possess suitable sensitising groups.

In the following sections, we highlight advances from the past five years, drawing on representative examples to illustrate the breadth of host molecules capable of selective and sensitive anion detection in aqueous and biological media.

## 4. Ln(iii) receptors for inorganic phosphate and nucleoside monophosphates

Phosphate is a key target for Ln(iii) probes due to its biological and environmental importance and strong binding to Lewis acidic Ln(iii) ions. In the human body, phosphate is essential for skeletal mineralisation, energy transfer, and cellular signalling, with blood concentrations tightly regulated between 0.8–1.45 mM.^75^ Elevated phosphate levels (hyperphosphatemia; >1.46 mM) are commonly observed in patients with chronic kidney disease and those undergoing dialysis, which can lead to vascular calcification and increased cardiovascular risk.^76^ The selective recognition of phosphate in aqueous media is therefore of considerable interest for diagnostic sensing, wherein receptors must discriminate phosphate from abundant competing oxyanions such as bicarbonate (23–29 mM) and lactate (0.5–1.0 mM).^29^ At physiological pH, phosphate exists mainly as H_2_PO_4_^−^ and HPO_4_^2−^ (∼1 : 4 ratio),^77^ requiring receptors to operate effectively in the millimolar range while minimising interference from these competing anions. In environmental systems, phosphate concentrations are typically much lower (0.01–0.1 mM) but have increased significantly in surface waters due to agricultural runoff and fertiliser overuse, leading to eutrophication, algal blooms, and aquatic dead zones.^78^ The wide disparity between biological and environmental phosphate levels underscores the need for receptors with finely tuned affinities and selectivity profiles suited to their intended application.

**Fig. 5:**
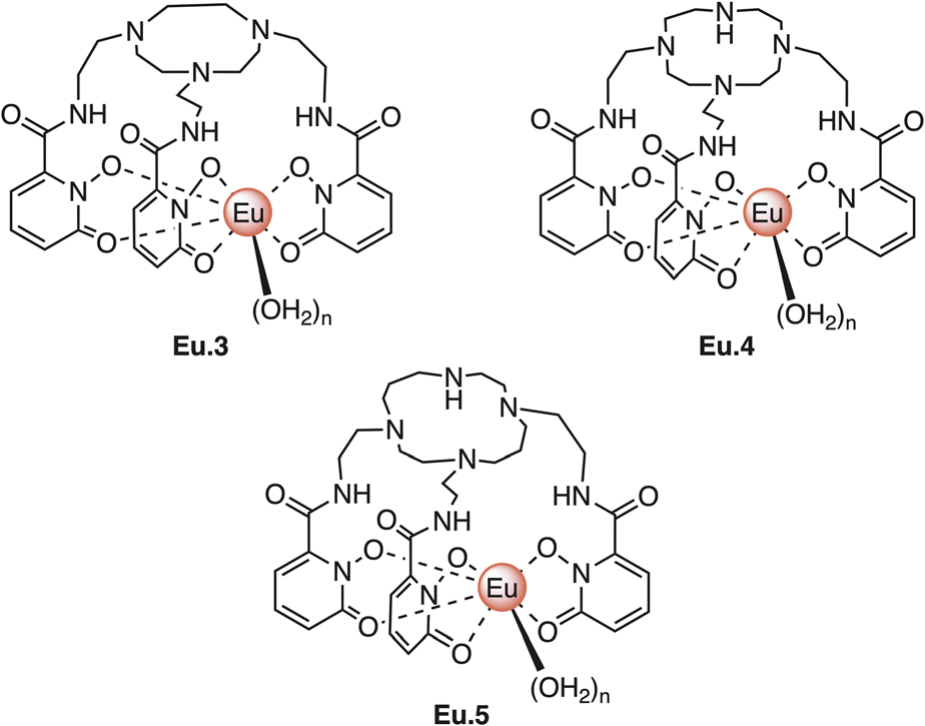
Tripodal Eu(iii) probes bearing different macrocyclic caps, developed by Pierre *et al.* for phosphate recognition.

Pierre and co-workers recently developed a series of inorganic phosphate receptors, each incorporating three 1,2-hydroxypyridinoate arms, capable of selectively extracting phosphate from aqueous media (Fig. 5).^79^ The Eu(iii) complexes differ in the nature of the macrocyclic cap, which contains either three or four nitrogen atoms and varies in ring size. The tris-bidentate ligands leave either two or three coordination sites available for water molecules. In the presence of ten equivalents of phosphate, complexes Eu.4 and Eu.5 showed displacement of two inner-sphere water molecules, consistent with coordination of two phosphate anions. In contrast, only one water molecule was displaced in the case of Eu.3. Interestingly, no clear correlation was observed between the number of inner-sphere water molecules and phosphate affinity, in line with their earlier observations on related tripodal systems.^80^ Cyclen-based complex Eu.4 displayed the highest affinity for phosphate (*K*_a1_ = 5.8 × 10^4^ M^−1^, *K*_a2_ = 7.4 × 10^4^ M^−1^) despite having only two inner-sphere water molecules, whereas complexes Eu.3 and Eu.5 have *q* = 3. Upon phosphate binding Eu.3 exhibited an emission lifetime of 0.24 ms, whereas Eu.4 and Eu.5 showed similar lifetimes of 0.40 and 0.39 ms, respectively. All three complexes had luminescence quantum yields (*Φ*_em_) in the range of 0.8–1% in water. All complexes demonstrated high selectivity for phosphate over potentially competing anions such as bicarbonate and chloride, which is particularly advantageous given their higher concentrations in biological media. The high selectivity of these tris-bidentate complexes for phosphate is impressive given the absence of steric hindrance at the open coordination sites and the absence of pre-organised hydrogen-bonding or electrostatic interactions.

**Fig. 6:**
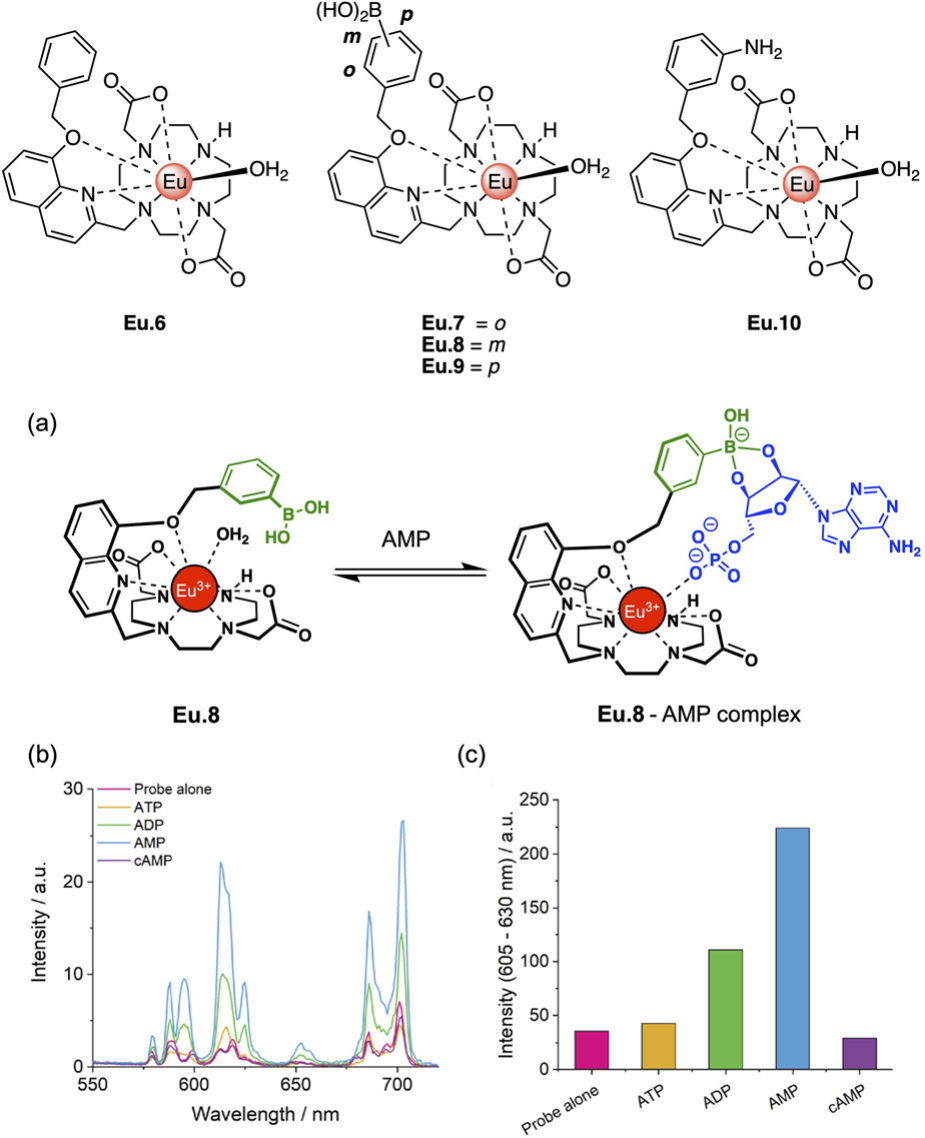
(Upper) Series of Eu(iii) probes developed by Butler and co-workers, including phosphate-selective probe Eu.6, boronic-acid functionalised regioisomers Eu.7, Eu.8, and Eu.9 for the selective recognition of AMP, and amine-functionalised Eu.10 for selective binding of PAP. (Lower) (a) Proposed binding mode of AMP to Eu.8, involving phosphate-Eu(iii) coordination and a boronate–diol interaction (b) emission spectra of Eu.8 showing selective enhancement with AMP over ATP, ADP, and cAMP (c) corresponding changes in the intensity of the Δ*J* = 2 emission band in the presence of 1 mM AMP, ATP, ADP, and cAMP, measured in 10 mM HEPES buffer (pH 7.0, 295 K). Adapted from ref. 43 with permission from the Royal Society of Chemistry, copyright 2022.

Butler and co-workers developed the monocationic complex Eu.6 which achieves highly selective phosphate recognition in water, by combining a rigid macrocyclic framework with a sterically demanding 8-(benzyloxy)quinoline pendant arm (Fig. 6).^43^ This chromophore coordinates the Eu(iii) centre in a bidentate manner, blocking the axial binding site and creating a cavity accessible only to the monodentate anions inorganic phosphate and AMP. Importantly, chelating oxyanions, including ATP, lactate and bicarbonate, are all sterically excluded, whereas inorganic phosphate binding induces a large and distinctive change in Eu(iii) emission. In 10 mM HEPES buffer (pH 7.0, 295 K), addition of 1 mM HPO_4_^2−^ produces an approximate 3-fold enhancement in overall Eu(iii) luminescence (*Φ*_em_ increasing from 1.7% to 5.3%), involving a large increase in the hypersensitive Δ*J* = 2 band around 614 nm. The binding constant was determined to be log *K*_a_ = 3.56 ± 0.01 under these conditions. Emission lifetime measurements in H_2_O (0.19 ms) *versus* D_2_O (0.25 ms) confirm displacement of the coordinated water molecule (*q* = 0), consistent with direct phosphate binding. Notably, the emission response is unchanged in the presence of excess bicarbonate, lactate, acetate, or sulfate, confirming that these anions are excluded by the sterically demanding ligand.

Building on this phosphate-selective scaffold, Butler and co-workers developed the complex Eu.8,^43^ incorporating a *meta*-phenylboronic acid moiety to enable multisite recognition of adenosine monophosphate (AMP) through reversible binding to the ribose *cis*-diol functionality (Fig. 6). AMP is a biologically important nucleotide involved in cellular energy metabolism and signal transduction (Fig. 4). Its recognition in water is highly challenging due to structural similarity with ADP and ATP, combined with its lower negative charge and lower abundance in biological systems.^81–84^ Addition of 1 mM AMP to Eu.8 in 10 mM HEPES buffer (pH 7.0, 295 K) induces an approximate 4-fold enhancement in overall Eu(iii) luminescence (from its initial *Φ*_em_ of 1.5%), including a prominent 6-fold increase in the Δ*J* = 2 band. In contrast, ATP causes only minor changes in emission and cAMP does not bind at all (Fig. 6b and c). The apparent binding constant for AMP is log *K*_a_ = 4.07 ± 0.01 under these conditions. ADP produces less than half the AMP response, reflecting both weaker binding of ADP to the Eu(iii) centre and greater quenching by water in the second coordination sphere. DFT calculations of the host-guest complex, Eu.8–AMP, indicate that simultaneous phosphate-Eu(III) coordination and formation of a cyclic boronate ester with the ribose sugar is geometrically feasible, supporting the proposed multisite binding mode (Fig. 6a). Luminescence lifetimes confirm displacement of coordinated water by AMP, consistent with coordination of its phosphate group to the Eu(iii) centre. Eu.8 successfully reverses the typical nucleoside phosphate selectivity profile (AMP > ADP > ATP) observed for most synthetic receptors,^81–84^ which was exploited to develop an *in vitro* assay for real-time phosphodiesterase activity, directly tracking AMP production without the need for labelled substrates, antibodies, or coupled enzymes.^43^

To establish the optimal geometry for AMP binding, Butler and co-workers subsequently developed two regioisomeric Eu(iii) complexes, Eu.7 and Eu.9, varying the position of the boronic acid on the benzyl capping unit.^50^ In 10 mM HEPES buffer at pH 7.0, Eu.9 also exhibits strong AMP binding (log *K*_a_ = 4.22), with a 6-fold enhancement in the Δ*J* = 2 emission band, while ATP produces negligible changes. However, discrimination between AMP and ADP is lower than for Eu.8 which remains the most selective. High-resolution mass spectrometry confirms 1 : 1 host–guest binding for AMP, including a stabilising boronate ester formation with the AMP ribose group. In contrast, Eu.7 shows no response towards any nucleoside phosphate, attributed to direct coordination of the *ortho*-boronate ester to the Eu(iii) centre which blocks the anion binding site. The quantum yield of Eu.7 was higher (*Φ*_em_ = 9.0%) than that of Eu.8 and Eu.9 (*Φ*_em_ = 1.5% and 2.0%, respectively) and *q* = 0 was determined for Eu.7, confirming direct coordination of the boronate ester. The emission lifetime of Eu.7 in water was 0.42 ms, whilst it was approximately half this value for both Eu.8 and Eu.9 (0.20 and 0.19 ms, respectively).

These results highlight the role of ligand conformation and boronic acid positioning, with Eu.8 providing the optimal geometry for selective AMP recognition in water. Notably, the *para*-boronic acid probe Eu.9 was subsequently utilised to monitor inorganic phosphate transport across lipid bilayers, facilitated by novel synthetic anion transporters developed by Valkenier and co-workers.^85,86^ Using this phosphate-sensitive Eu(iii) probe alongside ^31^P NMR spectroscopy, the synthetic transporter could mediate H_2_PO_4_^−^/Cl^−^ antiport, H_2_PO_4_^−^ uniport, and Cs^+^/H_2_PO_4_^−^ symport into vesicles, representing the first example of inorganic phosphate transport by a neutral synthetic receptor.

The design of AMP-selective probe Eu.8 provided an effective starting point for the selective detection of the nucleotide PAP (adenosine-3′,5′-diphosphate) over PAPS (adenosine-3′-phosphate-5′-phosphosulfate).^87^ PAP is the common by-product of biological sulfation reactions, in which sulfotransferase enzymes catalyse the transfer of a sulfate group from PAPS to diverse acceptor substrates (Fig. 4). Distinguishing PAP from PAPS is difficult owing to their close structural similarity: PAP contains two monophosphate groups, while PAPS possesses the same scaffold but bears an additional terminal sulfate. The ability to differentiate these anions is essential for monitoring sulfotransferase activity, yet simple, direct assays remain limited.^88,89^ To target PAP selectively, a *meta*-amino group was introduced onto the phenyl ring of the Eu(iii) receptor to give Eu.10 (Fig. 6), enabling a two-point interaction involving coordination of one phosphate to the Eu(iii) centre and hydrogen bonding between the second phosphate and the pendant amine.^87^ In contrast, the sulfate group of PAPS is less strongly attracted to the Lewis-acidic Eu(iii) ion, disfavouring binding. Eu.10 was able to selectively bind PAP in 50 mM TRIS buffer (pH 7.4, 295 K), producing a 1.8-fold higher emission intensity compared with PAPS. Binding constants of log *K*_a_ = 4.0 and 3.5 were determined for PAP and PAPS, respectively. The greater luminescence enhancement with PAP arises primarily from displacement of the inner-sphere water molecule, with additional contributions arising from suppression of photoinduced electron transfer (PeT) from the peripheral amine group. Eu10 displayed a low quantum yield in water (*Φ*_em_ = 0.3%) and relatively short emission lifetimes (*τ*H_2_O [unbound/PAP bound] = 0.05/0.14, *versus τ*D_2_O = 0.06/0.17), attributed to intramolecular PeT from the nitrogen lone pair on the aniline ring quenching the Eu(iii) excited state, combined with vibrational quenching by coordinated water in its unbound form. The selective emission response of Eu.10 towards PAP was exploited to produce the first direct, real-time assay of a heparan sulfate sulfotransferase activity, in microplate format.

These examples highlight that binding of inorganic phosphate and phosphate monoesters to Ln(iii) complexes is primarily driven by strong hard Lewis acid–base interactions between the Ln(iii) centre and phosphate oxygen donor atoms. However, selectivity requires additional ligand design elements beyond intrinsic metal–anion affinity. In the tris-bidentate tripodal systems, which possess open coordination sites and lack steric constraints, selectivity can be tuned through the peripheral electrostatic environment surrounding the metal centre. Incorporation of positively charged or hydrogen-bond donating groups around the capping unit enhances phosphate affinity through favourable second-sphere interactions, whilst negatively charged substituents positioned near the binding site can suppress anion recognition entirely. In contrast, macrocyclic systems based on cyclen utilise steric control around the Eu(iii) centre to help achieve selectivity. Bulky pendant arms restrict access to the coordination site, favouring monodentate binding of inorganic phosphate whilst sterically excluding larger chelating oxyanions such as ATP, ADP, or lactate. This ligand scaffold can then be extended towards nucleoside monophosphate recognition through incorporation of secondary binding interactions. For example, addition of a boronic acid functionality enables cooperative recognition of AMP through simultaneous phosphate coordination to Eu(iii) and reversible boronate ester formation with the ribose *cis*-diol, whilst pendant amine groups can promote selective PAP recognition through additional hydrogen-bonding interactions with a second phosphate moiety. Overall, these systems demonstrate that selective phosphate and phosphate monoester recognition can be achieved through a combination of intrinsic hard Lewis acid–base affinity, tuning of peripheral ligand electrostatics, steric control, and incorporation of secondary recognition elements.

## 5. Ln(iii) receptors for halides

Halide recognition in aqueous solution remains a significant challenge, particularly for chloride, the most abundant physiological anion involved in neuronal growth and central nervous system development. Understanding chloride transport is critical for diseases such as cystic fibrosis and epilepsy. While some highly selective synthetic chloride receptors function in organic solvents, for example the pioneering triazolo cage developed by Flood and co-workers, which binds chloride with ∼10^17^ M^−1^ affinity in dichloromethane,^90^ the situation is more challenging in water. The difficulty arises from chloride's high free energy of hydration (Δ*G*_hyd_ = 340 kJ mol^−1^), which necessitates strong electrostatic or metal–ligand interactions. This principle is exemplified in fluoride recognition using Ln(iii) complexes: despite fluoride's even higher hydration energy (Δ*G*_hyd_ = 465 kJ mol^−1^), its high charge density and Lewis basicity enables effective coordination to Ln(iii) centres. An early key example includes a cationic Eu(iii) complex reported by Charbonnière, in which fluoride is sequestered in a supramolecular Eu–F–Eu dimer (log *β* = 13.0 ± 0.3), stabilised by π–π stacking of the imidazole pendant arms.^37^ Another example is the Eu(iii) complex Eu.15 developed by Butler, which forms a 1 : 1 complex with fluoride in water with high selectivity over Cl^−^, Br^−^, I^−^, HPO_4_^2−^, CH_3_CO_2_^−^, HSO_4_^−^, and NO_3_^−^ (log *K*_a_ = 4.1 ± 0.1 in 25 mM MES, pH 6).^91^Eu.15 was subsequently utilised in anion transport experiments, where Eu.15 (Fig. 9) resides inside liposomes and reports on selective fluoride transport facilitated by synthetic anionophores, monitored using time-resolved emission spectroscopy.^92,93^ These examples illustrate how Ln(iii) complexes can overcome large hydration energies to achieve selective fluoride binding, providing a foundation for designing chloride-selective receptors in aqueous media.

**Fig. 7:**
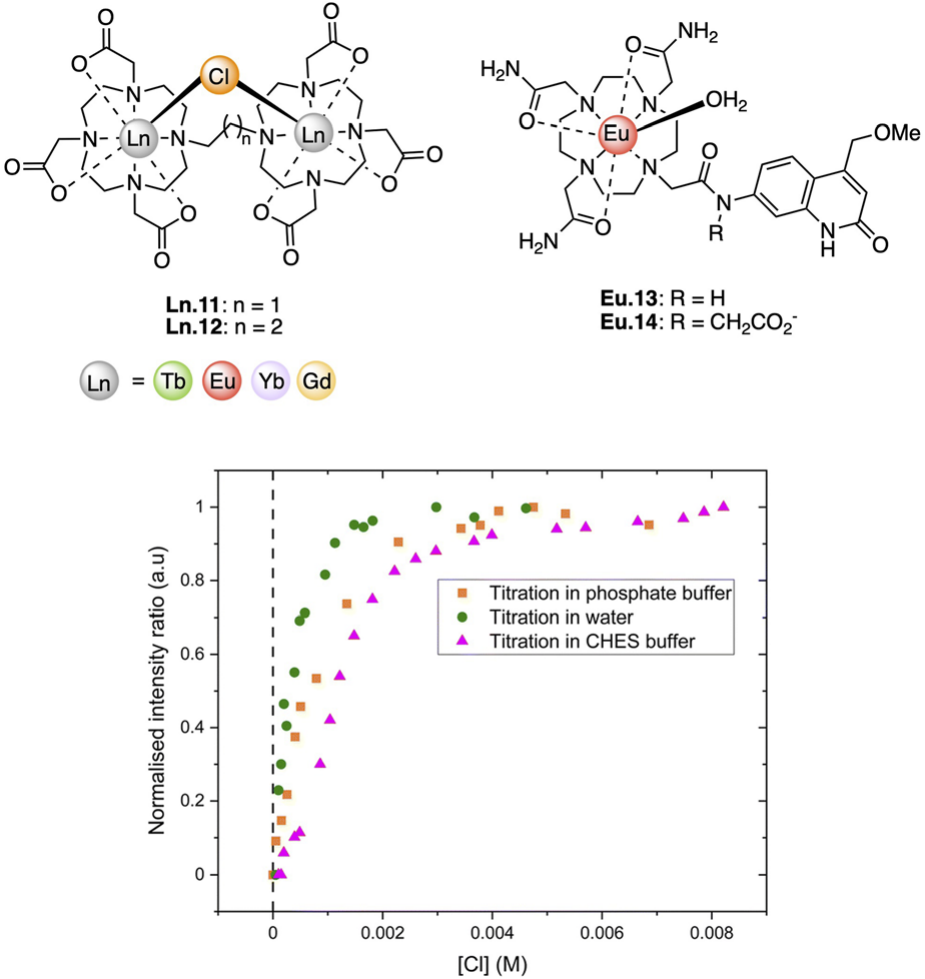
(Upper) Chloride-selective binuclear complexes Ln.11 and Ln.12 developed by Faulkner and co-workers, and carbostyril-bearing complexes Eu.13 and Eu.14 developed by Borbas *et al.*, capable of binding fluoride. (Lower) Chloride chelation by the binuclear complex Eu.11. Plot showing the normalised emission ratio of Δ*J* = 2/Δ*J* = 1 obtained from steady-state emission titrations for 1 mM Eu.11 against KCl in deionised water, 0.01 M phosphate buffer (pH 7.4), and 0.01 M CHES buffer (pH 9.9). Adapted from ref. 94 with permission from the Royal Society of Chemistry, copyright 2023.

Faulkner and co-workers recently reported a novel approach for chloride recognition using preorganised, binuclear and kinetically inert Ln(iii) complexes, Ln.11 and Ln.12, bridged by flexible ethane and propane linkers, respectively (Fig. 7).^94^ These overall neutral complexes create a preorganised binding pocket between two lanthanide centres, where chloride can bridge the Ln(iii) ions, enabling effective chloride recognition in water where mononuclear analogues show very weak binding. The binuclear design minimises conformational entropy loss upon binding, while the flexible spacer allows cooperative chloride coordination, overcoming intermetallic repulsions.

Chloride binding to Eu.11 and Eu.12 was quantified in water (*K*_a1_ = 2800 ± 7% and 4550 ± 3.9%) and was found to increase in 10 mM phosphate buffer at pH 7.4 (*K*_a1_ = 9400 ± 12.2% and 6640 ± 7.3%), suggesting competitive phosphate interactions, as evidenced by changes in the form of the emission spectra in phosphate buffer compared with water. Fig. 7a shows the change in emission ratio of Δ*J* = 2/Δ*J* = 1 of Eu.11 with increasing KCl in various aqueous media including deionised water, 10 mM phosphate buffer (pH 7.4), and 10 mM CHES buffer (pH 9.9). Fig. 7b shows the speciation model derived from titration data, revealing formation of the chloride-bridged host–guest complex. High-resolution ESI-MS confirmed the formation of the ternary chloride complexes. Eu.11 and Eu.12 also bind to fluoride in water, with the first binding event showing *K*_a1_ = 600 000 ± 39.7% and 10 480 ± 5.9%, respectively, followed by binding of a second and third fluoride ion at higher concentrations. Titrations with bromide and iodide indicated very weak or negligible binding, demonstrating high selectivity for chloride over these more lipophilic anions. Interestingly, no change in Eu(iii) luminescence lifetime was observed upon chloride binding, likely due to counteracting effects of PeT and intermetallic quenching, which oppose the expected lifetime increase from removal of O–H oscillators. These results highlight the potential of binuclear lanthanide complexes as selective chloride receptors in competitive aqueous media.

Borbas and co-workers synthesised a series of Eu(iii) and Tb(iii) complexes based on triazamacrocyclic and tetraazamacrocyclic ligands, each bearing a carbostyril antenna linked through a secondary or tertiary amide bond.^95^ Two representative tricationic Eu(iii) complexes Eu.13 and Eu.14 are shown in Fig. 7. The authors investigated how ligand structure, antenna orientation, and PeT quenching influence the recognition of fluoride and cyanide. The overall charge of the complexes was varied from +1 to +3, and the number of inner-sphere water molecules ranged from 1 to 3.

Addition of fluoride in 10 mM TRIS buffer at pH 8.1 produced substantial turn-on responses for several of the probes, with the largest increase (25-fold) observed for Eu.13, where *Φ*_em_ reached 7.2%. This enhancement arises from two complementary mechanisms: displacement of inner-sphere O–H quenchers increases the intrinsic Eu(iii) quantum yield, while suppression of antenna-to-metal PeT improves the sensitisation efficiency.^67^ Secondary amide-linked and more positively charged complexes exhibited larger increases in sensitisation, consistent with PeT being more prominent in more reducible Eu(iii) centres. In contrast, addition of cyanide quenched Eu(iii) luminescence in all cases (*Φ*_em_ = 0.05% for Eu.13), despite causing partial water displacement. This is likely due to interaction of CN^−^ with the antenna linker, possibly forming cyanohydrin adducts,^96^ which perturbs the electronic structure of the antenna and decreases sensitisation efficiency without significantly affecting the Eu(iii) coordination environment. These results highlight that both inner-sphere coordination and antenna-mediated PeT are important for anion-specific luminescence responses, and that changes in ligand structure can dramatically influence the balance between emission ‘turn-on’ and quenching effects.

In summary, halide recognition in aqueous solution by Ln(iii) complexes follows the general trend expected from hard Lewis acid–base interactions, with binding affinity decreasing from fluoride to chloride to iodide in line with decreasing basicity. Fluoride, as the hardest halide, binds relatively strongly to a range of Ln(iii) complexes and is able to compete effectively with coordinated water. However, despite this higher affinity, fluoride is typically present only at very low concentrations in biological systems and is therefore unlikely to compete significantly in chloride sensing applications. Instead, achieving selective chloride recognition in biological media remains challenging due to competition from other strongly coordinating oxyanions, such as phosphate and bicarbonate. As halides are small anions, steric and geometric organisation around the metal centre becomes particularly important for achieving selectivity. This can be achieved through cooperative multi-nuclear binding motifs, such as the chloride-bridged binuclear Ln(iii) complex reported by Faulkner and co-workers,^94^ or the supramolecular Eu–F–Eu sandwich complex reported by Charbonnière and co-workers.^37^ In contrast, mononuclear Ln(iii) complexes have been reported that show preferential fluoride binding but minimal response towards chloride in aqueous media.^91^ The development of a Ln(iii) receptor capable of selectively binding chloride in water, combined with a large and distinctive emission response, would therefore represent a significant advance for biological sensing applications.

## 6. Ln(iii) receptors for polyphosphate anions

**Fig. 8:**
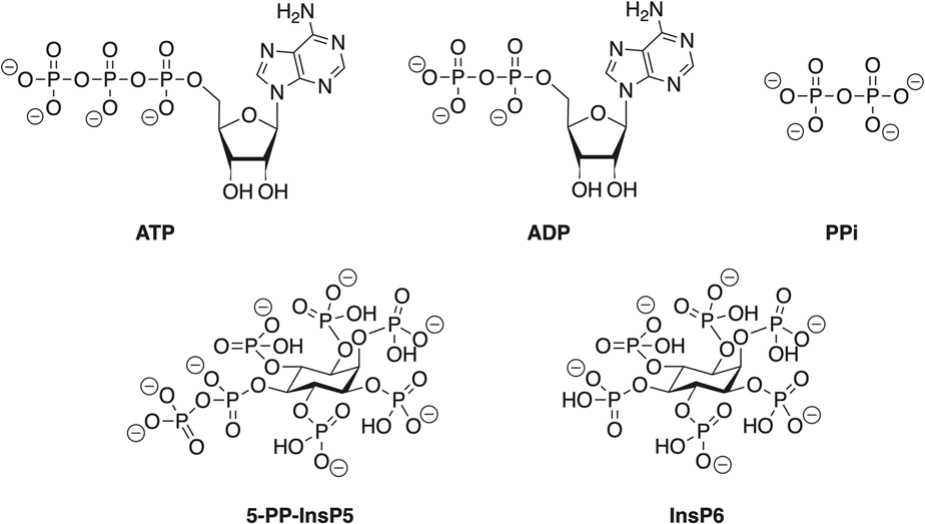
Structures of biologically important polyphosphate anions recognised by lanthanide(iii) probes.

Polyphosphates anions such as adenosine triphosphate (ATP), adenosine diphosphate (ADP), and pyrophosphate (PPi) are key targets for supramolecular detection due to their critical roles in cellular energy metabolism and signalling (Fig. 8).^9,82,97^ Enzymes such as kinases, which catalyse protein phosphorylation, and ATPases, which hydrolyse ATP to ADP, play essential roles in a range of cellular processes. Kinases in particular are frequently misregulated in diseases such as cancer, making them major targets for novel therapeutics.^98^ Reliable high-throughput screening methods are essential for identifying enzyme inhibitors, yet most current approaches rely on expensive and unstable antibodies, or require labelled substrates, and provide only end-point measurements that preclude real-time analysis.^99^

The majority of synthetic receptors have been developed to target ATP, taking advantage of its higher negative charge and higher concentrations in biological environments.^9,97^ However, these systems often suffer from interference from other polyphosphate anions such as PPi and ADP. Designing a receptor that selectively recognises ADP presents additional challenges, as the features that favour ATP recognition instead disadvantage ADP, due to its lower negative charge and lower biological abundance, meaning that competing species such as ATP and PPi can readily outcompete binding. For a Ln(iii) receptor to be effective, it should combine metal–ligand coordination with complementary non-covalent interactions, including hydrogen bonding or π–π stacking with the adenosine moiety, while also featuring a binding site whose size and geometry are matched to the diphosphate unit rather than the triphosphate of ATP.

**Fig. 9:**
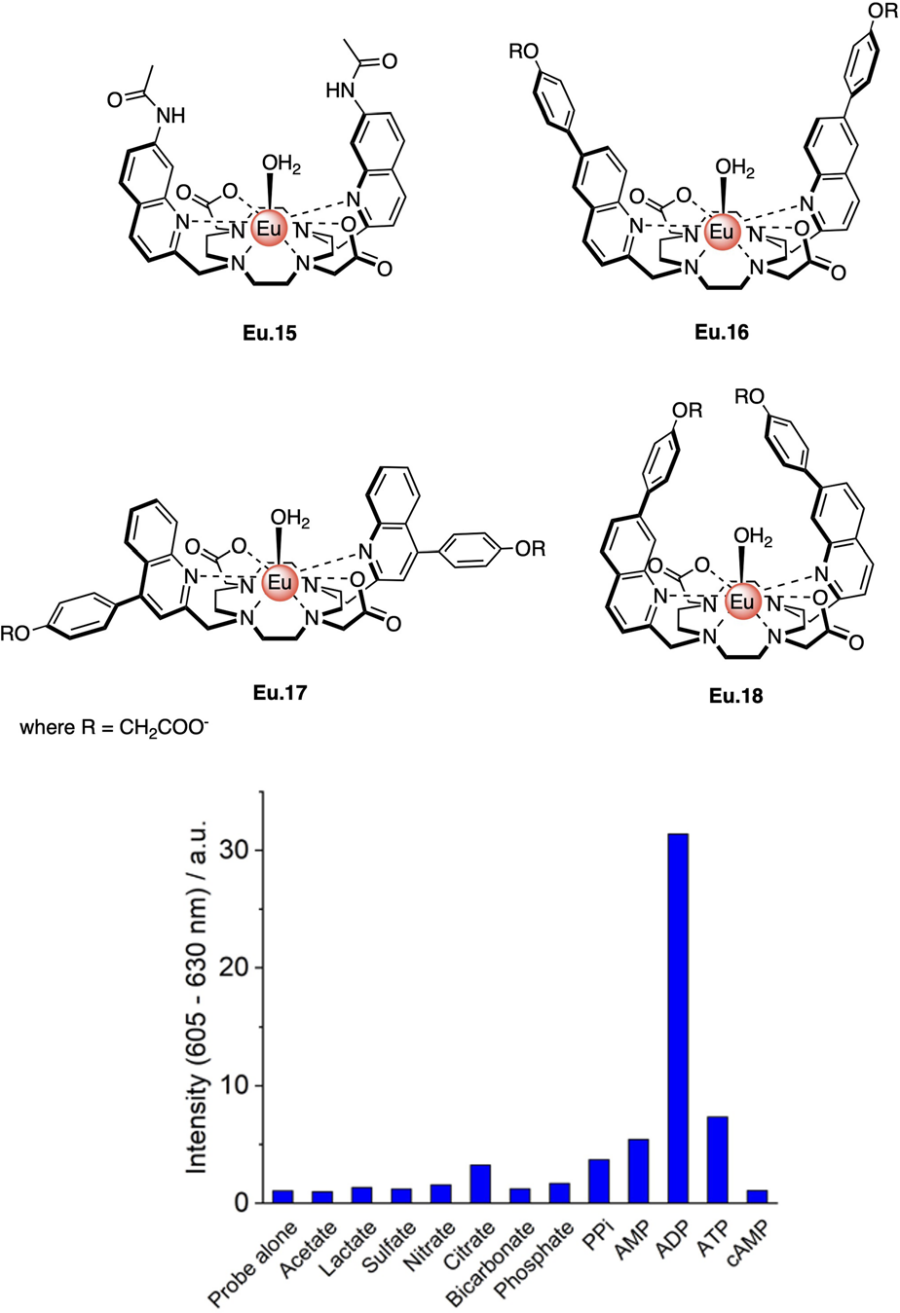
(Upper) Structures of the parent complex Eu.15, featuring an ADP-binding cavity formed by two quinoline antennae, and the regioisomeric complexes Eu.16–Eu.18 bearing π-conjugated arms, developed by Butler and co-workers for selective ADP recognition. (Lower) Bar chart displaying the emission enhancement of the Δ*J* = 2 (605–630 nm) band of Eu.16 with selected anions (1 mM each), measured in 10 mM HEPES at pH 7.0 and 295 K, *λ*_ex_ = 337 nm.

Earlier work by Butler and co-workers showed that the monocationic europium complex Eu.15 (Fig. 9) has sufficient flexibility to accommodate ADP within the cavity defined by the two quinoline amide arms.^59^ Upon binding ADP, the coordinated water molecule is released, causing a 7.5-fold enhancement in overall Eu(iii) emission intensity (from its initial *Φ*_em_ of 7.0%) and more than a doubling of the emission lifetime (from 0.48 ms to 1.18 ms). This complex was subsequently developed into a microplate assay enabling real-time monitoring of kinase-catalysed reactions *via* time-resolved detection of ADP formation.^100^ Despite the much larger emission enhancement observed for ADP compared with ATP, similar binding affinities and emission spectral shapes were obtained for these anions in water, particularly within the Δ*J* = 1 emission band (582–605 nm), indicating similar coordination environments at the Eu(iii) ion. However, because only the most emissive species is observed in the spectrum, the presence of more than one host–guest species could not be ruled out. ^31^P NMR of the Eu.15–ATP adduct provide strong evidence for bidentate binding of ATP *via* the α- and γ-phosphate groups (Fig. 2) whereas the ADP binding mode could not be determined due to exchange-broadened signals.^101^

To gain a clearer understanding of the binding mode, Eu L_3_-edge EXAFS and high-field EPR/ENDOR spectroscopy were subsequently used to characterise the interactions of ATP, ADP, and AMP with Ln.15 (Ln = Eu, Gd).^102^ ATP was shown to bind unambiguously in a bidentate 1 : 1 host–guest structure *via* the α- and γ-phosphate groups, consistent with the solution NMR data, and EXAFS analysis ruled out the possibility of a 2 : 1 host–guest complex. On the other hand, ADP was found to adopt both bidentate and monodentate binding modes in solution (Fig. 2), with a preference for bidentate coordination, which was not apparent from emission and NMR studies alone. AMP binds exclusively in a monodentate manner with weaker affinity, as expected. Integrating EXAFS and EPR with NMR and luminescence data can thus provide a more complete picture of host–anion binding, helping to guide the design of lanthanide complexes with improved selectivity.

**Fig. 10:**
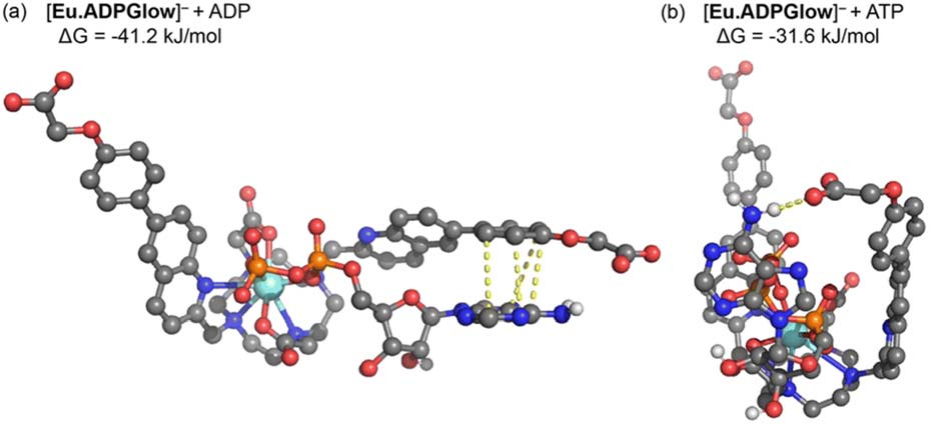
DFT optimised structures and binding free energies (Δ*G*) of Eu.16 (referred to as Eu.ADPGlow in the original paper^103^) bound to (a) ADP and (b) ATP. In part (a) dashed bonds indicate π–π stacking (3.45–3.92 Å) between the adenine base of ADP and the conjugated phenyl group of Eu.16. In contrast, no π–π stacking is observed in the ATP complex; instead, in part (b) shows a single hydrogen bond (1.97 Å) between the peripheral carboxylate of Eu.16 and the adenine base of ATP. Reproduced from ref. 103 with permission from the Royal Society of Chemistry, copyright 2025.

Building on this, the Butler group recently developed Eu.16, a switch-on Eu(iii) probe for highly selective detection of ADP in water.^103^ The probe incorporates two *trans*-related quinoline antennae now extended at the 6-position with π-conjugated phenoxyacetate groups, creating a deeper binding site that favours ADP. Selective binding of the diphosphate group to the Eu(III) centre, supported by π–π stacking between the adenosine moiety and the conjugated quinoline arm, drives the remarkable ADP selectivity, consistent with the DFT-optimised host-guest structure in Fig. 10a. In comparison, no such π–π stacking was observed in the DFT structure of the ATP-Eu.16 adduct (Fig. 10b). In 10 mM HEPES at pH 7.0, addition of 1 mM ADP produces a 33-fold enhancement in Eu(iii)-centred emission (*Φ*_em_ increases from 0.3% to 9%), whereas ATP, PPi, AMP, and other biologically relevant anions caused minimal (<2-fold increase) or no response at all. Notably, the probe is fully water-soluble and resistant to non-specific protein binding, which was an issue previously encountered for Eu.15. Complex Eu.16 was shown to permeate mammalian cells, distributing predominantly in the lysosomes. Future ligand designs could focus on achieving localisation within the mitochondria,^104^ thereby enabling real-time monitoring of ADP dynamics in a targeted subcellular environment.

In subsequent work, the Eu(iii) probe series was extended to evaluate how the geometry of the π-conjugated pendant arms affects anion selectivity. Two new complexes were synthesised with the same π-conjugated arms at the 4- and 7-positions of the quinoline scaffold, alongside the 6-substituted Eu.16.^105^ The 4-substituted complex Eu.17 possesses an open binding site and adopts a predominantly SAP configuration in solution, leading to binding of ADP (log *K*_a_ = 3.38 in 10 mM HEPES at pH 7.0) with limited selectivity over ATP or AMP. In contrast, the 7-substituted complex Eu.18 introduces significant steric hindrance at the Eu(iii) centre that effectively precludes all anion binding, as reflected by minimal emission changes even with excess ADP. Thus, the 6-substituted Eu.16 achieves an optimal balance of steric constraint and binding site accessibility, enabling highly selective ADP recognition through Eu(iii) coordination and π–π stacking. Consistent with these trends, NMR and X-ray data show that the analogous 4-, 6- and 7-methoxy substituted complexes display progressively greater TSAP character, while their emission lifetime measurements in methanol reveal decreasing solvent coordination, from 0.9 ms (4-substituted) to 0.7 and 0.5 ms (6- and 7-substituted complexes). Together, these trends help to rationalise why the 6-substituted Eu.16 achieves the optimum geometry and binding site accessibility for selective ADP binding.

**Fig. 11:**
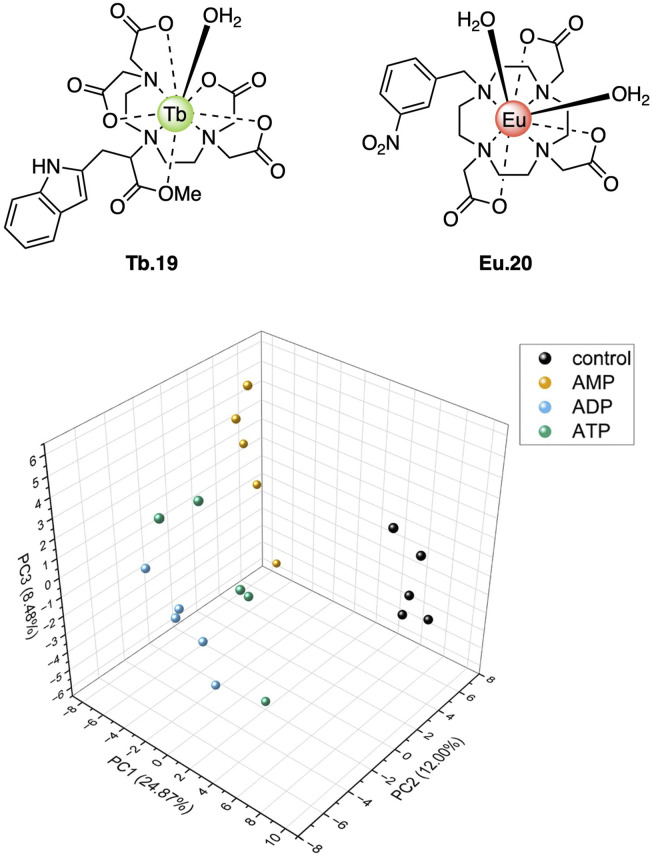
(Upper) Structures of complexes Tb.19 developed by Patra and Eu.20 developed by Hill, each capable of discrimination of nucleoside phosphates. (Lower) Principal component analysis of emission spectra of Eu.20 (control) and Eu.20 in the presence of AMP, ADP, and ATP (5–20 equivalents) measured in 0.1 M HEPES buffer. Reproduced from ref. 110 with permission from the Royal Society of Chemistry, copyright 2025.

Patra and co-workers developed a terbium(iii) complex Tb.19 based on an open-chain DTTA (diethylenetriamine-*N*,*N*,*N*′,*N*″-tetraacetic acid) ligand, bearing a pendant l-tryptophan antenna (Fig. 11).^106^ In 10 mM HEPES buffer at pH 7.2, efficient antenna-to-metal energy transfer populates the Tb(iii) excited state, giving rise to green emission that is selectively quenched in the presence of guanine nucleotides such as GMP, GDP and GTP. The hydration state of the complex remains unchanged (*q* = 1) in the presence of these anions, indicating that quenching occurs purely *via* outer-sphere interactions, involving weak but favourable π–π stacking between the guanine base and the indole ring of the antenna. This interaction enables photoinduced electron transfer from guanine to the excited indole, which suppresses energy transfer to Tb(iii) ion, resulting in diminished Tb(iii) emission. Stern–Volmer analysis reveals markedly larger quenching constants for guanine nucleotides (*K*_SV_ = 9.12–13.14 mM^−1^) compared with adenine analogues (0.65–0.70 mM^−1^), consistent with more efficient PeT arising from the higher HOMO energy of guanine and its more favourable electronic match with the indole antenna. The similar *K*_SV_ values across GMP, GDP, and GTP confirm that the selectivity is governed primarily by nucleobase-dependent PeT efficiency, rather than phosphate number or overall charge.

Array-based sensing strategies offer an alternative to highly selective receptor design by exploiting the differential, ‘cross-reactive’ responses of receptors toward structurally related analytes.^107–109^ In such approaches, discrimination arises from analyte-dependent response patterns that are analysed using multivariate statistical methods, most commonly principal component analysis (PCA) or linear discriminant analysis (LDA). Lanthanide complexes are very well suited for sensing arrays, as their emission spectra comprise multiple, well-resolved bands whose relative intensities and lifetimes are highly sensitive to changes in the local coordination environment. This richness in spectral and lifetime data can be effectively analysed by PCA. In earlier work, Butler and co-workers demonstrated this principle using a sensing array composed of four Eu(iii) and Tb(iii) receptors, which enabled discrimination between eight nucleoside phosphate anions (ATP, ADP, AMP, GTP, GDP, GMP, cAMP, and Pi) based on their differential luminescence responses.^101^

More recently, Hill and co-workers showed that PCA could be applied to the full emission spectrum of a single europium complex to distinguish three closely related nucleoside phosphates. Using the charge-neutral nitrobenzyl-containing DO3A complex Eu.20 (Fig. 11), they demonstrated clear discrimination between AMP, ADP, and ATP in 0.1 M HEPES buffer at pH 7.4.^110^ Analysis of the principal component loadings showed that discriminatory information was distributed across the entire europium(iii) emission profile, including typically overlooked bands such as Δ*J* = 3 (645–670 nm). Similar binding constants were determined for AMP and ADP (log *K*_a_ = 3.53 and 3.38, respectively), with ATP being approximately 10-fold higher (log *K*_a_ = 4.34). Similar emission lifetime values of 0.74, 0.87, and 0.76 ms were measured in the presence of excess AMP, ADP, and ATP, respectively (compared with 0.41 ms for Eu.20 alone). This study demonstrates that a single lanthanide probe contains sufficient information across its full emission spectrum to discriminate closely related phosphoanions when analysed using multivariate techniques, despite their similar binding constants and emission lifetimes.

Inositol pyrophosphates are highly charged signalling molecules involved in apoptosis, cell growth, and energy regulation.^64,111^ Among these, 5-PP-InsP_5_ (5-diphosphoinositol pentakisphosphate) is the most abundant second messenger in mammalian cells, with intracellular concentrations of 0.5–5 µM.^112^ 5-PP-InsP_5_ contains a pyrophosphate group at the 5-position, whereas the structurally related InsP_6_ (inositol hexakisphosphate), which is more abundant, contains six monophosphate groups and lacks a diphosphate (Fig. 8). A molecular probe that can distinguish 5-PP-InsP_5_ from InsP_6_ would enable new assays of inositol pyrophosphate metabolism and signalling. Butler, Potter and co-workers developed a macrocyclic Eu(iii) complex Eu.21 to achieve this selectivity.^113^ The complex features two neutral carbonyl amide donors, creating a highly electropositive metal centre that favours bidentate coordination of the pyrophosphate group, while the peripheral carboxylate groups ensure water solubility and minimise non-specific interactions with other anions, including InsP_6_.

**Fig. 12:**
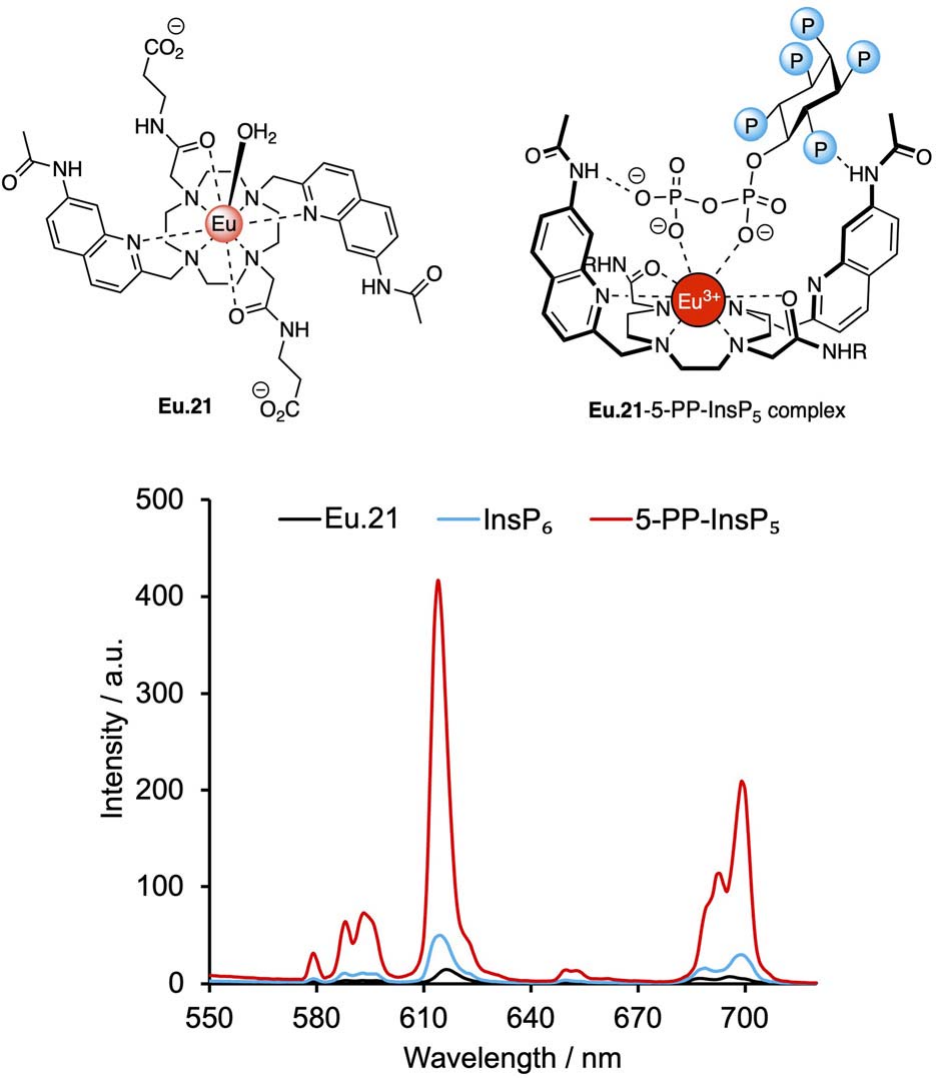
(Upper) Complex Eu.21 developed by Butler and co-workers and an illustration of the proposed binding mode between Eu.21 and the inositol pyrophosphate, 5-PP-InsP_5_. (Lower) Change in europium emission spectra of [Eu.3]^+^ (5 µM) in the presence of 250 µM 5-PP-InsP_5_ (red line) and InsP_6_ (blue line).

Addition of 5-PP-InsP_5_ to Eu.21 in 10 mM HEPES at pH 7.0 produced a 22-fold enhancement in Eu(iii) emission intensity, whereas InsP_6_ induced only a minor 3-fold increase (Fig. 12), reflecting a strong interaction between Eu.21 and 5-PP-InsP_5_ (log *K*_a_ ≈ 5.4). Under the same conditions, the emission lifetime of Eu.21 nearly doubled upon binding 5-PP-InsP_5_ (from 0.56 to 1.0 ms), whereas little change was observed in the presence of excess InsP_6_ (0.59 ms). Bidentate binding of the pyrophosphate group of 5-PP-InsP_5_ to the Eu(iii) ion was supported by DFT calculations and NMR studies and is the structural basis for the unique emission enhancement, since the diphosphate is the only feature distinguishing 5-PP-InsP_5_ from InsP_6_. Notably, Eu.21 can detect 5-PP-InsP_5_ with low micromolar sensitivity and without interference from InsP_6_ or other common cellular inositol and carbohydrate phosphates. This selective response was exploited in the development of a label-free, time-resolved assay to monitor the enzymatic conversion of 5-PP-InsP_5_ to InsP_6_, providing a practical tool to study inositol pyrophosphate metabolism and associated signalling pathways.^113^

As shown in these examples, selective recognition of polyphosphates by Ln(iii) complexes requires a balance between strong metal–phosphate coordination and ligand geometries capable of accommodating larger, conformationally flexible polyphosphate motifs. In contrast to monophosphate recognition, where sterically demanding macrocyclic scaffolds can effectively drive selectivity for anions such as AMP (see Section 4), receptors for ATP, ADP, PPi, and inositol pyrophosphates must accommodate larger and chelating binding modes. Several effective systems have been developed based on cyclen-based macrocycles bearing *trans*-related antennae, in which partial dissociation or conformational flexibility of the pendant donor arms enables chelation of polyphosphate anions at the Ln(iii) centre. Within these systems, steric hindrance around the coordination sphere is clearly important for controlling selectivity between closely related polyphosphates. For example, increasing steric demand around the Eu(iii) centre can disfavour binding of larger triphosphates such as ATP whilst permitting selective coordination of ADP, whereas excessive steric hindrance can suppress anion binding entirely.

Beyond direct metal coordination, secondary interactions such as π–π stacking with the nucleobase can further stabilise binding and enhance selectivity, as observed between Eu.16 and ADP. Peripheral electrostatic tuning of the Ln(iii) complex also plays an important role, with negatively charged substituents helping to reduce non-specific interactions with highly charged interferents and favour selective recognition of target polyphosphate binding modes. This is exemplified by the selective recognition of 5-PP-InsP_5_ over InsP_6_ by Eu.21, where peripheral carboxylate groups minimise non-specific electrostatic interactions with InsP_6_ whilst maintaining strong binding of the pyrophosphate motif of 5-PP-InsP_5_.^113^

## 7. Ln(iii) receptors for baicarbonate and α-hydroxycarboxylates

Bicarbonate (HCO_3_^−^) is a biologically important anion involved in cellular pH regulation, metabolic waste transport, and renal function. Disruption of bicarbonate homeostasis, frequently linked to mutations in bicarbonate transporters, is associated with diseases such as renal disorders, haemolytic anaemia, and glaucoma.^114^ These roles have motivated the development of synthetic receptors and responsive probes for HCO_3_^−^, particularly systems capable of reporting its spatio-temporal dynamics in biological environments. Seminal studies by Parker and co-workers showed that cationic europium(iii) complexes bearing azaxanthone-based sensitisers can bind bicarbonate selectively in water and biological samples.^64,111^ Reversible association of HCO_3_^−^ resulted in displacement of two inner-sphere water molecules and a marked enhancement in Eu(iii) emission, especially in the hypersensitive Δ*J* = 2 transition. On the basis of spectroscopic and structural evidence, the anion was proposed to coordinate in a bidentate manner (Fig. 2), consistent with Parker's earlier work on carbonate chelation to lanthanide centres.^14^

**Fig. 13:**
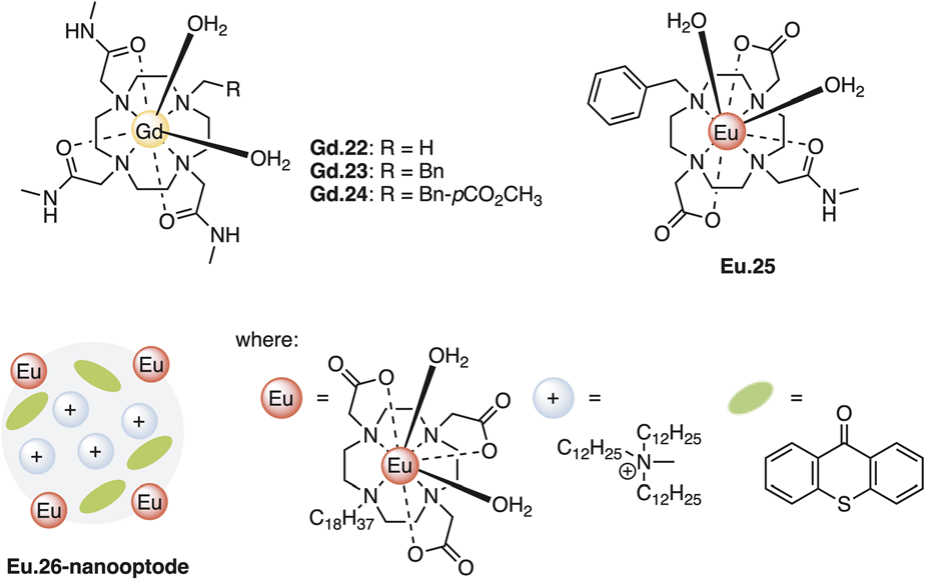
Gd(iii) complexes Gd.22–Gd.24 developed by Pierre and co-workers for selective capture of bicarbonate. Complex Eu.25 and Eu.26-nano-optode developed by Sørensen and co-workers with affinity for bicarbonate.

Recently, a series of macrocyclic gadolinium complexes developed by Pierre and co-workers reveal how geometric control at the metal centre can overturn the usual basicity-driven preference of lanthanides for phosphate over bicarbonate.^44^ Phosphate typically binds more strongly to Ln(iii) complexes because of its higher basicity, but selective HCO_3_^−^ recognition can be achieved by enforcing a coordination geometry that disfavours monodentate phosphate while preorganising the metal site for the bidentate chelation mode of bicarbonate. Binding studies across a series of DO2A and DO3A-derived complexes (including representative complexes Gd.22–Gd.24, Fig. 13) showed that the most effective designs incorporate three neutral amide donors (giving a tricationic complex), and a peripheral benzyl group that subtly distorts rather than blocks the anion binding pocket, and two inner sphere water molecules. The lead complex, Gd.23, contains three amide pendant arms and a benzyl substituent on the fourth macrocyclic nitrogen, widening the O–Gd–O angle defined by the two inner-sphere water molecules to match the bite angle of HCO_3_^−^. This geometry provides relatively high-affinity, selective bicarbonate binding (*K*_a_ = 2.8 × 10^4^ M^−1^ in 50 mM TRIS at pH 8.0), which is matched to ocean-relevant concentrations where [HCO_3_^−^] ≈ 2.4 mM. Notably this complex design suppresses direct coordination of phosphate or chloride. Importantly, the Gd(iii) complex releases bicarbonate (in the form of H_2_CO_3_) under mildly acidic conditions (pH ≈ 5), enabling full regeneration of the Gd(iii) receptor and CO_2_ recovery. This work establishes Gd.23 and related complexes as the first lanthanide-based receptors suitable for practical bicarbonate capture in direct ocean capture (DOC) technologies.

Sørensen and co-workers previously synthesised a structurally related monocationic europium(iii) complex, Eu.25 (Fig. 13), based on a DO2A scaffold with one carbonylamide donor, a single benzyl unit near the Eu(iii) centre to increase lipophilicity at the anion binding site, and two coordinated water molecules.^115^ In contrast to Pierre's bicarbonate-focused studies, this work targeted biologically relevant α-hydroxycarboxylates, such as lactate and citrate, an important class of metabolites that has featured prominently in the development of lanthanide-based probes.^38,116,117^ Anion binding studies were conducted in MES-buffered DMSO/water at pH 5.8 to suppress pH-dependent emission changes, and prevent competitive bicarbonate binding arising from dissolved CO_2_ at higher pH. Addition of α-hydroxycarboxylates (l-lactate, l-malate, citrate), and phosphate displaced the coordinated water, resulting in increases in Eu(iii) emission with minimal changes to the spectral shape and thus crystal field.^115^ Lifetimes were used to compare binding strength, with steeper slopes in the lifetime isotherms indicating stronger interactions. The benzyl-modified site enhanced binding of the α-hydroxycarboxylates, and the results showed that the charge of the complex primarily serves as a modulator for the residual positive charge on the Eu(iii) ion, strengthening the lanthanide-centred interaction. The study illustrates how lipophilicity and residual positive charge at the Eu(iii) centre can be modulated for recognition of metabolically relevant oxyanions.

In subsequent work focused on bicarbonate sensing, Sørensen and co-workers prepared Ln(iii)-doped polystyrene nano-optodes using a modular strategy in which a C18-functionalised Eu.DO3A complex (Eu.26) provided the luminescence readout, while the bulky lipophilic cation TDMA^+^ and the antenna chromophore thioxanthone acted as auxiliary components (Fig. 13).^118^ By preparing four nanoparticle formulations, including Eu.26 alone (NP1), Eu.26 with TDMA^+^ (NP2), Eu.26 with antenna (NP3), and Eu.26 with both TDMA^+^ and antenna (NP4), they showed how lanthanide incorporation, surface charge modulation, and intermolecular antenna sensitisation collectively influence the optical response toward biologically relevant oxyanions.

The nano-optodes respond to bicarbonate in 20 mM HEPES buffer at pH 7.4, through binding at the Eu.26 centre in NP1 and NP3, which displaces inner sphere water and suppresses non radiative decay, leading to longer Eu(iii) lifetimes (from approximately 0.6 ms to 0.75 ms). Despite the lifetime increases, the emission intensity decreases because bicarbonate binding increases the Eu.26 to antenna distance in NP3, and the intermolecular antenna effect is strongly distance dependent; reduced sensitisation therefore outweighs the reduced vibrational quenching of the Eu(iii) excited state. In contrast, the positively charged nano-optodes NP2 and NP4 show non-specific responses: TDMA^+^ creates an electropositive surface that attracts bicarbonate and partially displaces Eu.26 from the hydrophobic surface, leading to shorter lifetimes and lower emission intensity due to increased solvation and reduced antenna sensitisation. Competition studies support these observations and are consistent with previous reports on related Eu.DO3A complexes.^119^ Neutral NP3 shows binding of lactate, weaker binding of hydrogen phosphate, and no response to chloride, whereas charged NP4 exhibits only small, non-specific effects from chloride and lactate and a modest response to phosphate. Overall, the results show that antenna mediated sensitisation controls the signal magnitude, the anion binding mode primarily controls the lifetime changes, and nanoparticle surface charge determines whether the response to bicarbonate is specific or non-specific.

Overall, these studies highlight several key geometric and electrostatic factors governing Ln(iii) recognition of bicarbonate and α-hydroxycarboxylates. Both classes of anions preferentially coordinate the Ln(iii) ion through chelation, with bicarbonate forming relatively constrained four-membered rings, while α-hydroxycarboxylates such as lactate and citrate form five-membered chelate rings *via* cooperative binding of the carboxylate and α-hydroxyl groups (Fig. 2). Consequently, the O–Ln–O bite angle and overall coordination geometry play a decisive role in determining affinity and selectivity; Ln(iii) receptors that preorganise the binding site to match these bite angle requirements can therefore achieve enhanced selectivity, even against more basic competing oxyanions such as phosphate (as demonstrated with Gd.23). It should be noted that bicarbonate can bind, albeit weakly, to a wide range of Ln(iii) probes, which is an important consideration in the design of selective receptors. This background interaction is difficult to eliminate entirely due to the small size of the anion and its pH-dependent concentration in aqueous media, arising from equilibrium with dissolved CO_2_. Among α-hydroxycarboxylates, citrate typically binds more strongly than lactate due to its higher overall negative charge and increased electrostatic attraction to the Ln(iii) centre. As demonstrated originally by Parker and co-workers,^38^ selectivity can be tuned towards lactate by employing more sterically demanding Ln(iii) complexes in combination with negatively charged peripheral groups that disfavour binding of the more highly charged citrate.

## 8. Ln(iii) receptors for urate, ascorbate, and protein carboxylate residues

Electron-rich aromatic anions, such as urate and ascorbate, are important bioactive species in human physiology. Uric acid is the end-product of purine metabolism and is present in serum as the sodium salt at 0.13–0.46 mM and in urine at 1.5–4.5 mM.^120^ Uric acid serves as a key scavenger of reactive oxygen species. Ascorbate functions as both an antioxidant and pro-oxidant to maintain redox homeostasis, with concentrations ranging from near zero in erythrocytes to approximately 10 mM in neurons.^121^ Despite their central roles in metabolic and genetic processes, reliable and simple optical probes for monitoring these small bioactive anions in real-time, either *in vitro* or *in vivo*, remain scarce.

**Fig. 14:**
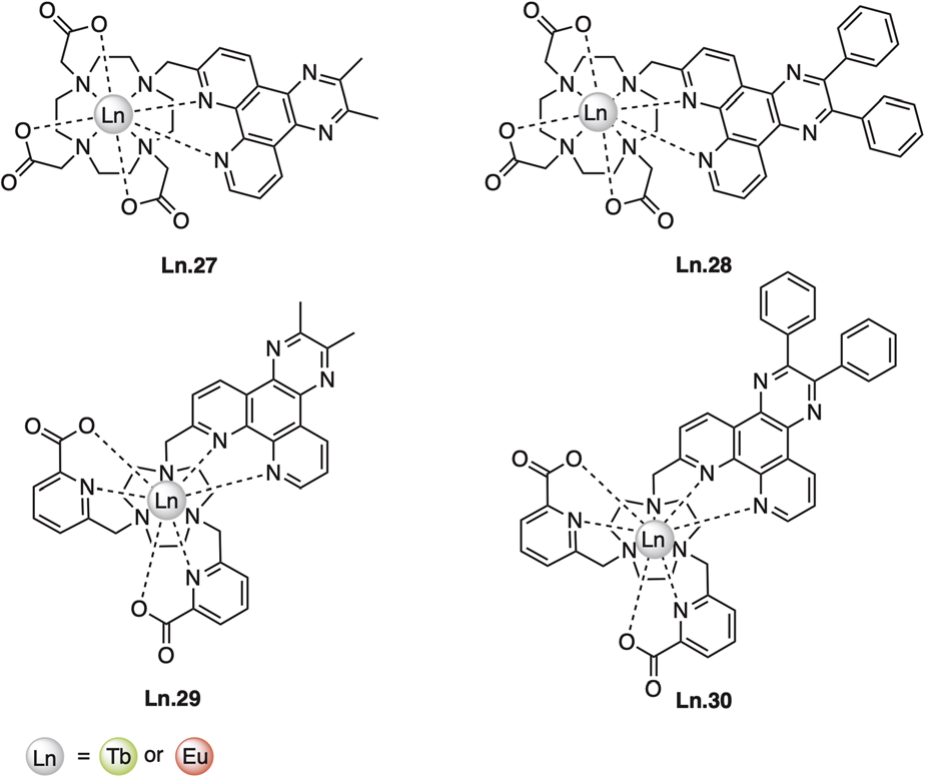
Series of lanthanide complexes Ln.27–Ln.30 developed by Parker and co-workers bearing tetra-azatriphenylene sensitisers for selective sensing of urate.

To address this challenge, Parker and co-workers developed a series of nonadentate ligands (Fig. 14) incorporating electron-poor tetra-azatriphenylene (dpqPh_2_ or dpqMe_2_) chromophores into cyclen and TACN scaffolds.^122^ These ligands provide a rigid, well-defined coordination environment that excludes water from the primary coordination sphere, avoiding vibrational quenching of the lanthanide excited state. Based on their earlier work, electron-rich species such as urate, ascorbate, and catecholates were shown to efficiently deactivate lanthanide excited states in systems bearing electron-poor chromophores (dpq/dpqC or azaxanthone).^70,71^ This quenching was hypothesised to occur *via* formation of a transient exciplex: a non-covalent excited-state complex between the ligand triplet state and the quenching species. Because the ligand triplet state lies closer in energy to the Tb(iii) ^5^D_4_ excited state than to the Eu(iii) ^5^D_0_ state, triplet depopulation *via* exciplex formation competes more effectively with Tb(iii) sensitisation. As a result, Tb(iii) complexes are more susceptible to quenching, providing the basis for ratiometric sensing, in which the ratio of Tb/Eu emission intensities can be used to detect urate in aqueous solutions.

Quenching studies conducted in 0.1 M HEPES at pH 7.4 showed that Eu.29 (*Φ*_em_ = 14%) and Tb.29 (*Φ*_em_ = 30%) are the most sensitive pair towards urate, with Stern–Volmer constants of 20 µM and 3 µM, respectively. A calibration curve of the Tb/Eu (540/614 nm) emission ratio *versus* urate concentration was effective in the 0–50 µM range. Temperature-dependent measurements showed that emission intensity of Eu.29 increased with temperature in the presence of urate, consistent with the entropically disfavoured exciplex formation, whereas iodide and ascorbate caused a decrease in emission with increasing temperature, indicating thermally activated collisional quenching. Further, ionic strength studies demonstrated that increased salt concentration weakens non-covalent exciplex formation, reducing urate-mediated quenching, whereas quenching by iodide and ascorbate followed the opposite trend. These results establish that transient exciplex formation underlies urate sensing and provide a mechanistic framework for designing selective lanthanide-based probes for its ratiometric detection in aqueous and biological environments.

**Fig. 15:**
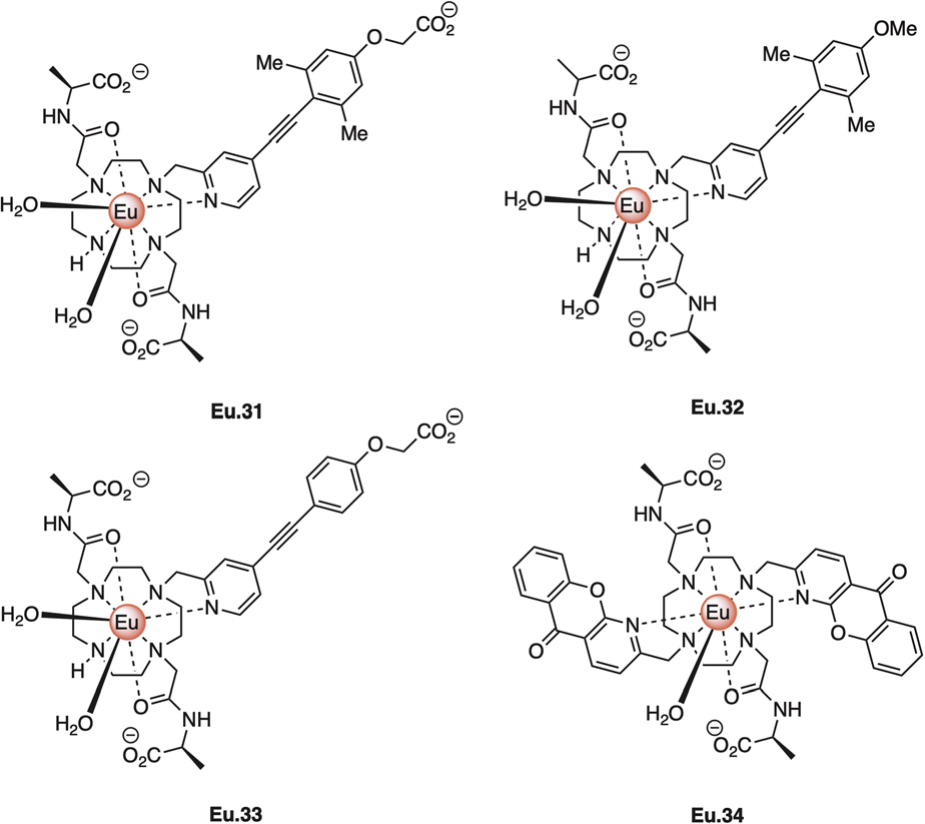
Series of Eu(iii) complexes Eu.31–Eu.33 developed by Parker and co-workers, each bearing a single aryl–alkynyl–pyridyl chromophore and alanine-derived pendent arms, for selectively protein binding and CPL sensing, compared with their previously reported complex Eu.34.

Glutamic acid and aspartic acid residues are important carboxylate recognition domains in proteins, capable of coordinating metal centres or engaging in electrostatic and hydrogen-bonding interactions. Certain chiral europium(iii) complexes exploit these anionic sites, in which displacement of coordinated or second-sphere water molecules reduces vibrational quenching, leading to enhanced emission intensity and excited-state lifetime. For example, Parker and co-workers recently developed a series of neutral di-aqua complexes (Fig. 15), each featuring a single aryl–alkynyl–pyridyl chromophore flanked by two *trans*-related chiral alanine-derived arms.^51^ The overall neutral complex Eu.31 binds selectively to human serum albumin (HSA) drug site 1 (DS-1), producing a 23-fold increase in luminescence intensity, an 87% increase in the Δ*J* = 2/Δ*J* = 1 emission ratio, and an increase in excited-state lifetime (from 0.28 to 0.38 ms), with a binding constant of log *K*_a_ = 6.67 (measured in 10 mM HEPES buffer at pH 7.4).

**Fig. 16:**
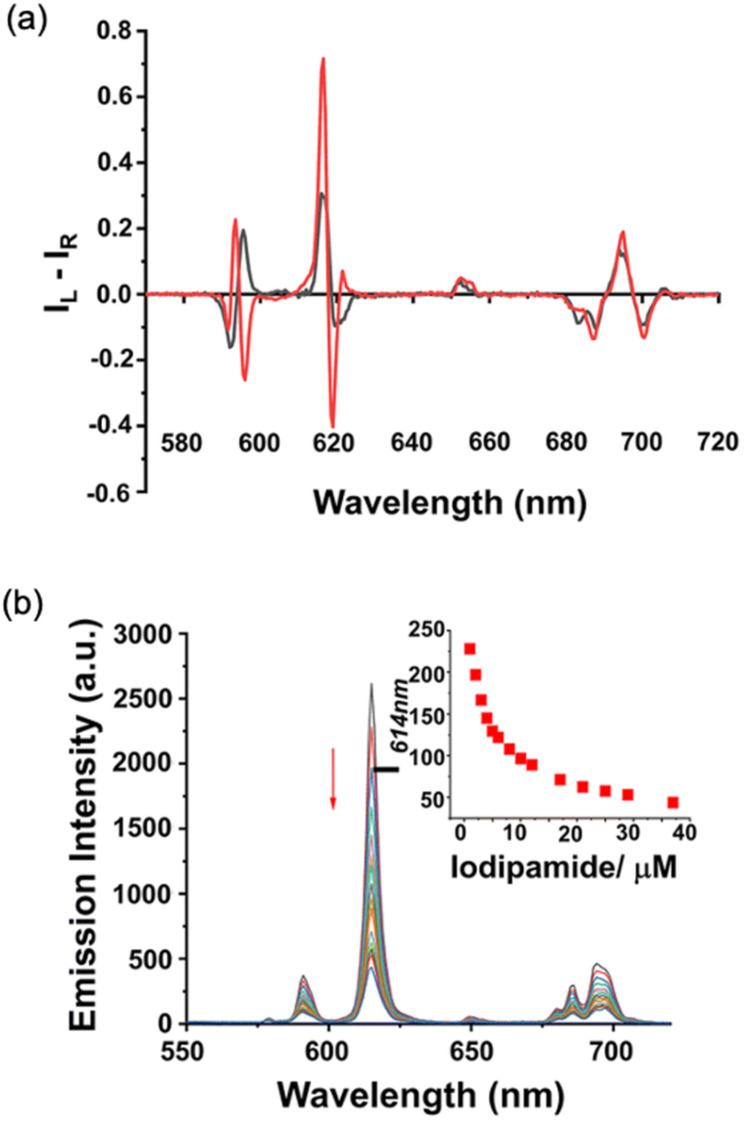
CPL studies of protein binding and competitive drug binding assay using Eu.31. (a) Enhancement of the CPL spectra of Eu.31 (black) upon addition of HSA (red) (5 µM complex, 35 µM HSA, *λ*_ex_ 340 nm, H_2_O, 298 K). (b) Decrease in the observed emission of Eu.31 (10 µM) upon competitive displacement from HSA (5 µM) by increasing iodipamide concentration (10 mM HEPES buffer, pH 7.4). Reproduced from ref. 51 with permission from the Royal Society of Chemistry, copyright 2025.

Importantly, binding of Eu.31 is stereoselective, favouring one helical diastereomer, and triggers a “switch-on” of circularly polarised luminescence (CPL) as shown in Fig. 16a. CPL arises from the differential emission of left- and right-circularly polarised light by a chiral emitter, in this case the Eu(iii) complex, and is quantified as the intensity difference (*I*_L_–*I*_R_) or *via* the luminescence dissymmetry factor (*g*_lum_). In Fig. 16a, the CPL spectrum is plotted as (*I*_L_–*I*_R_) *versus* wavelength, such that the sign and magnitude of the signal directly reflect the handedness and extent of chiral emission generated upon binding of Eu.31 within the chiral binding pocket of HSA.

Competitive titration with iodipamide, a dicarboxylate known to bind most strongly to DS-1 (log *K*_a_ = 6.99), confirms specific occupancy of the protein binding site and demonstrates that Eu.31 is tuned to probe sub-micromolar drug binding events (Fig. 16b). Analogous protein specific binding behaviour is observed for the cationic complex Eu.32 with α1-acid glycoprotein (α1-AGP), for which log *K*_a_ = 6.13 was determined. In contrast, weaker binding is observed for either complex with bovine serum albumin (BSA), and the less bulky complex Eu.33 exhibits weak, non-selective interactions with all serum proteins. These results highlight the importance of both size and charge complementarity in conferring protein specificity in lanthanide–protein interactions.

Molecular dynamics simulations provide mechanistic insight into this selectivity. The aryl–alkynyl–pyridyl moiety of Eu.31 inserts into the hydrophobic pocket of DS-1, while electrostatic and cation–π interactions with nearby Lys, Arg, and other charged residues further stabilise binding. The carboxylate side chain of Glu-188, positioned near the Eu(iii) centre, can reversibly coordinate the metal ion, causing displacement of bound water and further lowering the free energy of binding. Simulations capture both the initial intermediate binding geometry and the subsequent Glu-188 coordination, helping to explain the enhanced emission and activation of the CPL signal. Together, these results illustrate how cooperative hydrophobic and carboxylate interactions enable highly selective lanthanide binding to protein recognition sites, demonstrating the potential of Eu(iii) complexes as sensitive probes of local anionic and chiral environments in proteins.

In summary, it has been shown that electron-rich aromatic anions such as urate and ascorbate can be sensed through quenching of Ln(iii) emission, caused by interactions with the sensitising antenna rather than direct binding at the Ln(iii) centre, in contrast to many of the oxyanion receptors discussed herein. In these systems, emission quenching is attributed to non-covalent association with electron-poor aromatic chromophores, enabling transient exciplex formation between the ligand triplet state and the anion. This provides an efficient pathway for excited-state deactivation, particularly in Tb(iii) complexes where the antenna triplet state is energetically well matched for competitive depopulation. By contrast, protein recognition by Ln(iii) complexes can be achieved by coordination of glutamate and aspartate residues directly to the Ln(iii) centre, with displacement of bound water. This is supported by hydrophobic insertion of the antenna arm into defined protein binding pockets. It should be noted that Ln(iii) complexes bearing hydrophobic aromatic arms can also engage in non-specific protein interactions through hydrophobic contacts, meaning that selectivity for specific anionic residues is achieved only when steric complementarity and spatial positioning of the Ln(iii) centre relative to the binding site are enforced, as demonstrated by Parker and further rationalised through DFT and molecular dynamics simulations.

## 9. Conclusions

Luminescent lanthanide(iii) complexes represent an important class of receptors for reversible anion recognition in aqueous and biologically relevant media. In most reported systems, Ln(iii)–anion coordination provides the primary driving force for binding, complemented by careful control over ligand architecture, the steric environment, and overall complex charge to confer additional anion selectivity. It is increasingly clear that selectivity is governed not only by electrostatic and Lewis acid–base interactions, but also by the geometric and steric complementarity of the anion with the binding site, including the size and rigidity of chelate rings formed by multidentate oxyanions and the accommodation of monodentate guests at sterically demanding metal centres.

Sophisticated receptor designs have demonstrated that secondary interactions, such as hydrogen bonding, π–π stacking, and diol recognition motifs, can be integrated alongside Ln(iii) coordination to achieve higher levels of discrimination between closely related anions. These strategies have enabled advances in the selective sensing of nucleoside phosphates, carboxylates, and physiologically relevant inorganic anions such as phosphate and chloride. Crucially, anion binding can be translated into sensitive luminescent responses by deliberately modulating photophysical pathways: displacement of inner-sphere O–H (or N–H) oscillators enhances the intrinsic lanthanide quantum yield, while suppression or enhancement of ligand-to-metal energy transfer modulates the sensitisation efficiency. This mechanistic control underpins changes in emission intensity, lifetime, and spectral shape, with Eu(iii) in particular offering high sensitivity to perturbations in the local coordination environment, enabling high levels of anion discrimination in biologically relevant media.

Looking forward, several exciting directions are emerging. Chiral anion recognition at lanthanide centres enables enantioselective sensing and discrimination *via* circularly polarised luminescence, an approach further driven by recent advances in microscopy instrumentation.^123,124^ The growing use of sophisticated DFT and excited-state modelling can provide molecular-level insight into host–guest binding geometries and emission spectral changes, helping to guide the design of improved sensors. The development of heterometallic or multinuclear systems, particularly those linking Eu(iii) and Tb(iii) components, holds promise for ratiometric detection methods with enhanced reliability in complex biological media. Together, these advances underpin the development of lanthanide(III)-based anion receptors with improved selectivity and sensitivity, enabling background-free optical sensing and imaging in complex biological media with clear translational potential.

## Author contributions

The review article was written by S. J. B. with support by C. S.

## Conflicts of interest

There are no conflicts to declare.

## Data Availability

No primary research results, software or code have been included and no new data were generated or analysed as part of this review.
